# Regional variation in healthcare spending and mortality among senior high-cost healthcare users in Ontario, Canada: a retrospective matched cohort study

**DOI:** 10.1186/s12877-018-0952-7

**Published:** 2018-11-01

**Authors:** Sergei Muratov, Justin Lee, Anne Holbrook, Andrew Costa, J. Michael Paterson, Jason R. Guertin, Lawrence Mbuagbaw, Tara Gomes, Wayne Khuu, Jean-Eric Tarride

**Affiliations:** 1 Department of Health Research Methods, Evidence, and Impact, McMaster University, Hamilton, ON Canada; 2 Programs for Assessment of Technology in Health (PATH), The Research Institute of St. Joe’s Hamilton, St. Joseph’s Healthcare, Hamilton, ON Canada; 3 Division of Geriatric Medicine, Department of Medicine, McMaster University, Hamilton, ON Canada; 4 Division of Clinical Pharmacology and Toxicology, Department of Medicine, McMaster University, Hamilton, ON Canada; 5 Geriatric Education and Research in Aging Sciences Centre, Hamilton Health Sciences, Hamilton, ON Canada; 6 Institute for Clinical Evaluative Sciences (ICES), Toronto, ON Canada; 7 Center for Health Economics and Policy Analysis (CHEPA), McMaster University, Hamilton, Canada; 8 Département de Médecine Sociale et Préventive, Faculté de Médecine, Université Laval, Quebec City, QC Canada; 9 Centre de recherche du CHU de Québec, Université Laval, Axe Santé des Populations et Pratiques Optimales en Santé, Québec City, QC Canada; 10 Biostatistics Unit, Father Sean O’Sullivan Research Centre, St Joseph’s Healthcare, Hamilton, ON Canada; 11 Institute of Health Policy, Management and Evaluation, University of Toronto, Toronto, ON Canada; 12 Leslie Dan Faculty of Pharmacy, University of Toronto, Toronto, Canada; 13 Li Ka Shing Knowledge Institute, St. Michael’s Hospital, Toronto, ON Canada

**Keywords:** Senior high-cost users, Small area variation, Healthcare expenditures, Mortality

## Abstract

**Background:**

Senior high cost health care users (HCU) are a priority for many governments. Little research has addressed regional variation of HCU incidence and outcomes, especially among incident HCU. This study describes the regional variation in healthcare costs and mortality across Ontario’s health planning districts [Local Health Integration Networks (LHIN)] among senior incident HCU and non-HCU and explores the relationship between healthcare spending and mortality.

**Methods:**

We conducted a retrospective population-based matched cohort study of incident senior HCU defined as Ontarians aged ≥66 years in the top 5% most costly healthcare users in fiscal year (FY) 2013. We matched HCU to non-HCU (1:3) based on age, sex and LHIN. Primary outcomes were LHIN-based variation in costs (total and 12 cost components) and mortality during FY2013 as measured by variance estimates derived from multi-level models. Outcomes were risk-adjusted for age, sex, ADGs, and low-income status. In a cost-mortality analysis by LHIN, risk-adjusted random effects for total costs and mortality were graphically presented together in a cost-mortality plane to identify low and high performers.

**Results:**

We studied 175,847 incident HCU and 527,541 matched non-HCU. On average, 94 out of 1000 seniors per LHIN were HCU (CV = 4.6%). The mean total costs for HCU in FY2013 were 12 times higher that of non-HCU ($29,779 vs. $2472 respectively), whereas all-cause mortality was 13.6 times greater (103.9 vs. 7.5 per 1000 seniors).

Regional variation in costs and mortality was lower in senior HCU compared with non-HCU. We identified greater variability in accessing the healthcare system, but, once the patient entered the system, variation in costs was low. The traditional drivers of costs and mortality that we adjusted for played little role in driving the observed variation in HCUs’ outcomes. We identified LHINs that had high mortality rates despite elevated healthcare expenditures and those that achieved lower mortality at lower costs. Some LHINs achieved low mortality at excessively high costs.

**Conclusions:**

Risk-adjusted allocation of healthcare resources to seniors in Ontario is overall similar across health districts, more so for HCU than non-HCU. Identified important variation in the cost-mortality relationship across LHINs needs to be further explored.

**Electronic supplementary material:**

The online version of this article (10.1186/s12877-018-0952-7) contains supplementary material, which is available to authorized users.

## Background

High-cost health care users (HCU), a minority of individuals who consume a large proportion of health care resources, are a diverse group [1]. Due to their high burden on the healthcare system, a better understanding of various segments of the HCU population is needed to develop evidence-informed health care policy [1, 2]. In particular, seniors (patients 65 years of age and older), who account for about 15% of the population in the province of Ontario, account for approximately 60% of the total costs incurred by all HCU in the province [3–5]. Further, nearly half of senior HCU each year are incident cases [6, 7]. These “new” cases represent a stratum of the HCU population that can potentially be a target of preventative interventions and management, but they have not been adequately studied, especially in the context of regional variation.

Large geographical disparities in health care services have been documented globally [8, 9]. In Canada, marked regional variation has been identified in key healthcare services such as hospitalization [10], surgical procedures [11, 12], and use of prescription drugs [13, 14]. In contrast to this evidence of disparities in individual healthcare services, there is little information on variation in healthcare spending in the Canadian provincial context. While reports on regional variation in healthcare spending, especially the Medicare costs, have dominated the US political debate for more than a decade [15–19], only one Canadian study (British Columbia[BC]) has investigated regional variation in healthcare expenditures and found it to be modest [20]. While very informative, the BC study was not intended to investigate seniors specifically, not to mention senior HCU. Moreover, except for the total healthcare spending, the BC study did not provide information on variation among individual cost components such as hospitalization and physician costs which limits our understanding of the processes of care that contribute to higher or lower variation [21].

Understanding regional variation in health services utilization, costs and health outcomes can inform health services planning for senior patients, including senior HCU, in several ways. First, it allows planners to explore potential drivers of variation that deserve attention by describing the distribution of patient and care characteristics across health districts [22, 23]. Second, evidence suggests that planning and implementing health services with an “equity lens” can improve equity in resources allocation [24] and healthcare services use [25–27], and reduce regional variation in outcome distribution [28]. Third, measuring the relationship between costs and health outcomes among health regions is critical for policy makers to identify geographical “pockets” of efficient care (areas with lower spending and better outcomes). Recent studies have reported the level of inefficiency in Canada at 20% [29] with significant variations across Canadian provinces [30]. Moreover, even though available evidence of healthcare regional variation and efficiency has led policy makers to entertain the idea of cutting reimbursement rates in higher-spending regions [15, 31] or to establish new provider-physician integrated entities with spending benchmarks (accountable care organizations) [32], there is still a gap in our knowledge as to how regional disparities in healthcare spending affect regional patterns of health outcomes [33]. The lack of evidence on geographical variation in health outcomes seems to have contributed to the gap [34, 35].

To better inform decision and policy making in Ontario and fill a gap in the literature, the objectives of this study were: 1) to estimate regional variation in healthcare costs (total and by cost categories) and mortality among incident senior HCU compared to senior non-HCU; and 2) to examine the relationship between health spending and mortality by health districts for senior incident HCU compared to senior non-HCU.

## Methods

### Study design

A retrospective population-based matched cohort study was conducted using province-wide linked administrative data. More details on the study population and data sources are published elsewhere but are briefly summarized below [36].

### Study population

We generated a cohort of all incident senior HCU in the province of Ontario. This cohort was defined as consisting of seniors (aged ≥66 years) with annual total healthcare expenditures within the top 5% threshold of all Ontarians in the 2013 Ontario government fiscal year (FY2013) (i.e. incident year), and not in the top 5% in the 2012 fiscal year (FY2012).The threshold of 5% to define HCU is aligned with previous Canadian studies of this population [3, 7, 37, 38].The incident HCU cohort was matched to a cohort of non-HCU using a 1:3 matching ratio, without replacement based on age at cohort entry (+/− 1 month), sex and residence (based on Local Health Integration Networks [LHIN]). The “non-HCU” cohort was defined as those whose annual total health care expenditures in the 2012 and 2013 fiscal years were both below the financial threshold of the top 5% of all Ontarians in the respective year.

### Data sources

The patient-level dataset was created using 19 health administrative databases [36]. These datasets were linked using unique encoded identifiers and analyzed at the Institute for Clinical Evaluative Sciences (ICES) [39]. Health care expenditures were calculated using a person-level health utilization costing algorithm [40]. Total healthcare expenditures were comprised of 12 separate health service cost categories. Hospital costs were the sum of costs associated with inpatient care and same-day surgery. Physician costs were the sum of fee-for-service billings and capitation payments. Costs reported in this study are based on patients’ geographic location of residence. Costs are expressed in 2013 Canadian Dollars.

### Geographic unit of analysis

We used LHINs, Ontario’s regional health districts, as the geographic unit of analysis. Ontario’s 14 LHINs are responsible for the funding, planning and management of hospital- and community-based health services delivered to all residents within their geographic boundaries [41]. Services covered by the LHINs include most of hospital and community care such as inpatient care, long-term and home care, community mental health, rehabilitation and hospices among others [42], but exclude physician services, which are funded from a separate envelope.

### Variables

The study population was described at baseline (i.e., the year before HCU incident status), including comparisons of socio-demographic determinants (age, sex, residence, low income), health status (degree of morbidity, proportion of chronic conditions) and health system factors (e.g., number of physicians in the circle of care and whether a geriatrician was visited) between HCU and matched non-HCU. Subjects with low income status were identified based upon net household income reported to receive public drug benefit subsidy in FY2012. Compared to census-based neighborhood income measures, the Ontario Drug Benefit (ODB)-based low income status is a better reflection of personal income, as it relies upon actual net income. For a small proportion of HCU (3%) and non-HCU (13%) who did not fill a prescription in FY2012, low-income status was defined as census neighborhood income quintile 1. Rurality was defined using the Rural Index of Ontario (RIO): an ordinal measure ranging from 0 (urban) to 100 (rural) that considers population density and travel time to the nearest health facility [43].

Several measures were employed to describe health status. Level of morbidity was measured using Johns Hopkins Aggregated Diagnosis Groups (ADGs) that are derived from Johns Hopkins Adjusted Clinical Groups (ACGs): a person-focused, diagnosis-based way to measure patients’ illness [44]. In addition, the proportions of patients with prior malignancy and mental health conditions were computed using John Hopkins Expanded Diagnosis Clusters (EDCs). Finally, the proportions of patients with chronic obstructive pulmonary disease, congestive heart failure, diabetes, and rheumatoid arthritis were estimated using ICES-derived, validated chronic disease cohorts [45, 46].

### Outcomes

Several outcomes were assessed. HCU rate for each LHIN was defined as the number of senior HCU over the total number of seniors residing in the LHIN per 1000 population. Mean per capita total healthcare expenditures and mean per capita health expenditures for each care category were calculated as the costs incurred in the incident year over the total population in the HCU and non-HCU cohort. Finally, mortality for each cohort was defined as the prevalence of all-cause death within the incident year.

### Statistical analysis

Descriptive statistics (counts [%]; mean [SD] or median [Q1, Q3]) were summarized for baseline individual characteristics and outcomes. Characteristics of subjects in both HCU and non-HCU cohorts were compared using absolute standardized differences (SDD). SDDs of more than 0.1 are considered to indicate meaningful differences between the cohorts [47]. To describe the variation between the LHINs in terms of costs and outcomes, the coefficient of variations (CVs) defined as the ratio of the standard deviation to the mean were determined.

Because the calculated intra-class correlation coefficient (ICC) pointed toward statistically significant clustering within the LHINs across most of the cost components and mortality in both cohorts (Additional file 1), we fitted a mixed effects model to estimate between-region variation and risk-adjust for age, sex, the number of ADGs per patient, and low-income status for both costs and mortality. Compared to the CV or other summary statistics characterizing regional variation, these models provide additional information, including the between-LHIN variance estimate and the proportion of the observed variation explained by patient characteristics.

We specified the following general equation (Additional file 2):

$$ {\mathsf{y}}_{\mathsf{ij}}=\left({\mathsf{Beta}}_{\mathsf{0}}+{\mathsf{u}}_{\mathsf{0}\mathsf{j}}\right)+\sum {\mathsf{Beta}}_{{\mathsf{ij}}^{\ast }}{\mathsf{X}}_{\mathsf{ij}}+{\mathsf{e}}_{\mathsf{ij}}; $$

where *yij* – the outcome (costs or mortality) in patient i from LHIN_j_; *Beta*_*0*_ - the provincial mean; *u*0*j* – the random effect for each LHIN that is assumed *u*0*j*~*N*(0, *σ*2*u*); *Beta*_*ij*_ - the fixed effects of individual level characteristics; *Xij* - the vector of covariates at the individual level; *eij* - the residual error.

This type of models assumes that the mean outcome value for each LHIN vary randomly according to a normal distribution (*u*0*j*~*N*(0, *σ*2*u*)) whereas the effect of the patients’ covariates is fixed among the LHINs. The main interest in this type of analyses is the random effect (*u*0*j*) which characterizes variation between LHINs, where *σ*2*u* is the direct estimate of the variance.

For the mortality analyses, logistic regression was conducted by fitting generalized linear mixed models (GLMM) according to the general model specification provided above. To model healthcare expenditures, two methods were used based on the proportion of zero costs values in the data. Zero cost values arise when healthcare resources are not consumed (e.g. no contact with healthcare system or no hospitalization). For healthcare categories with no zero costs values GLMM were used. In the presence of zero costs values in the data, Hurdle mixed models were used to account for zeros [48, 49]. Hurdle models, also referred to as a two-part model, assumes that costs are generated by two statistically different processes. A binomial distribution (part 1) was used to determine whether any costs were incurred, and a gamma distribution (part 2) was employed to model positive costs (instances when costs> 0) [49–51]. Expected costs resulting from Hurdle models are then calculated by multiplying the probability of observing a cost by the value of the costs when observed. LHIN-specific random effects were incorporated into each part of the model to estimate between-LHIN variation in the probability of incurring any costs (*σ*2*u*1) and variation in costs once they were incurred (*σ*2*u*2), resulting into two random effects values. Similarly, the fixed effects associated with the individual level characteristics were included in each part of the Hurdle model.

Unadjusted and risk adjusted models were compared using both the likelihood ratio test (LRT) that follows a chi-squared distribution (*p*-value has to be less than 0.05) and information criteria (e.g., Akaike and Bayesian: lower values equal better fit) [52–54]. Statistical significance of coefficients was considered at alpha = 0.05. We also compared the observed data with predicted values by LHIN to investigate model adequacy. The coefficient of determination R^2^ was calculated to measure the proportion of the observed regional variation in outcomes explained by the covariate for each model (please see Additional file 2) [55, 56]. To assess uncertainty around the estimates of the random effects, we generated a bootstrap 95% confidence interval (CI) from the bootstrap sample of 1000 by looking at the 2.5th and 97.5th percentiles in this distribution.

To determine whether certain regions are more efficient than others, we examined the relationship between total healthcare spending (positive costs) and mortality in both cohorts. Building on an approach previously employed for hospital profiling [57, 58], the risk-adjusted random effects for total costs and mortality were first ordered from the smallest to the largest and then graphically presented together in a cost-mortality plane, one for each cohort. LHINs located at the left bottom quadrant of the plots (where the X axis represents mortality and the Y axis costs) are more efficient than others provided that the CI of random effects for both total costs and mortality does not cross 0. Analyses were conducted using SAS software version 9.4 (SAS Institute Inc., Cary, NC, USA). The NLMIXED procedure was used to fit all the models. To visualize HCU rates between LHINs, a heat map was created using QGIS (Quantum geographic information system, https://qgis.org).

## Results

### Baseline characteristics

We included 703,388 subjects (HCU = 175,847, non-HCU = 527,541). The HCU were similar to non-HCU with respect to age, sex, the proportion residing in urban centres, and the number of low-income subjects (Table 1). However, compared to non-HCU, HCU tended to have a higher number of comorbidities, and a larger proportion of subjects with a malignancy, common chronic diseases, and mental health issues. HCU were dispensed a higher number of prescription drugs, had more physicians involved in their circle of care, and were seen by a geriatrician more often. Additional file 3 provides more information on the variation in these characteristics between the 14 LHINs.

**Table 1:** Patient baseline individual and care characteristics, pre-incident year

Characteristic	HCU	Non-HCU	SDD
	Mean (SD)	Mean (SD)	
Age: subgroup (%)			
66–74	39.7 (2.5)	39.7 (2.5)	0.00
75–84	39.9 (0.8)	39.9 (0.8)	0.00
≥ 85	20.5 (2.5)	20.5 (2.5)	0.00
Sex (F, %)	52.7 (1.3)	52.7 (1.3)	0.00
Rurality (urban, %)	61.8 (27.6)	62.7 (28.0)	0.03
Low income senior (%)	17.6 (5.2)	16.8 (5.7)	0.01
Number of ADGs (mean)	10.1 (0.4)	7.9 (0.3)	0.50
Malignant neoplasms (%)	32.2 (2.6)	23.4 (3.2)	0.20
Common chronic conditions* (%)	60.6 (2.4)	44.8 (2.3)	0.30
Mental health# (%)	37.6 (4.1)	26.9 (2.5)	0.20
Number of MDs involved in care (mean)	8.1 (0.5)	5.6 (0.3)	0.50
Seen by a geriatrician (%)	2.8 (1.3)	1.1 (0.5)	0.12
Number of prescription drugs (mean)	8.4 (0.4)	5.6 (0.3)	0.60
Acute inpatient care (%)	3.8 (1.1)	2.1 (0.7)	0.10

ADGs- Aggregated Diagnosis Groups; HCU- high-cost user; *ICES- derived common chronic conditions (either one of the following: CHF-congestive heart failure; COPD- chronic obstructive pulmonary disease; DM- diabetes, MI- myocardial infarction, RA- rheumatoid arthritis); LHIN – Local Health Integrated Network; SD- Standard Deviation; # includes any of mental health conditions among Expanded Diagnosis Clusters (PSY01–12); SDD – absolute standardized difference; SDD ≥ 0.1 indicates a meaningful difference

### HCU rate

**Fig. 1:**
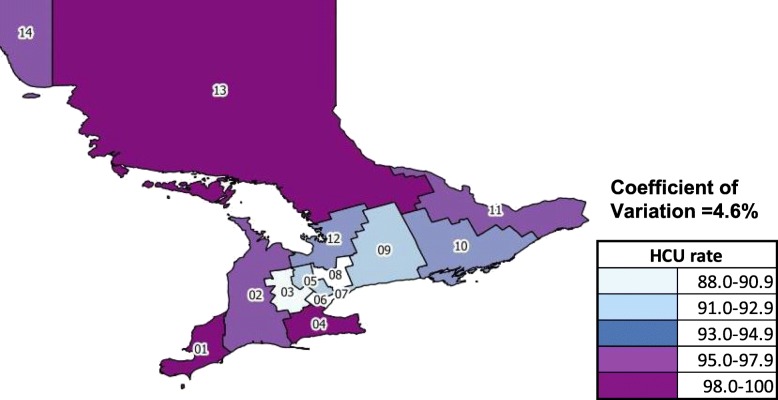
HCU rate, per 1000 seniors. 01- Erie St. Clair; 02- South West; 03- Waterloo Wellington; 04- Hamilton Niagara Haldimand Brant; 05- Central West; 06- Mississauga Halton; 07- Toronto Central; 08- Central; 09- Central East; 10- South East; 11- Champlain; 12- North Simcoe Muskoka; 13- North East; 14- North West

Figure 1 shows the distribution of HCU among the 14 health regions. The size of LHINs’ senior HCU population ranged from a low of 88.1 per 1000 seniors (Central 08 LHIN) to a high of 100.2 per 1000 seniors (North East 13 LHIN).One of the northernmost regions of Ontario (North East 13 LHIN), and two health regions in the southwest of the province (Erie St. Clair 01 LHIN and Hamilton 04 LHIN) had the highest rates of HCU in the province, whereas health regions in close proximity to Toronto tended to exhibit the lowest rates. Overall variation in HCU rates across the province had a CV of 4.6%.

### Unadjusted and adjusted costs and mortality by LHIN

The mean total 1-year observed costs per individual were $29,646 CAD and $2452 CAD for HCU and non-HCU, respectively. Hospital admissions represented the largest cost component among incident HCU accounting for 48.2% of the total costs. In non-HCU, prescription drugs were closely followed by physician costs as the top contributors to the total expenditures at 38.9% and 35.8%, respectively, while hospitalization accounted for 10.2%. All-cause mortality during the incident year among HCU was 13.6 times greater that of non-HCU (104.2 vs. 7.7 per 1000 seniors, respectively). Additional file 4 presents the observed and adjusted mortality and costs (total healthcare expenditures and its components) as well as the associated CVs. As shown in the tables, there was a very good agreement between observed and adjusted mean values across the mortality and cost components in both cohorts suggesting a good fit of the data. CVs for total costs and mortality were 3% and 6.8% for the HCU cohort, indicating little variation between LHINs. Higher CV values were observed for several cost components such as complex continuing care (CV of 45.1%), rehabilitation (CV of 7.2%), and dialysis (CV of 36.2%), and CVs were higher for the non-HCU matched cohort. In all analyses that converged, the models incorporating patient individual covariates were preferred to the models without covariates as shown by the LRT tests and lower AIC and BIC values. For non-HCU, the two-part mixed effects models did not converge in 4 cost components (mental health, long-term care, complex continuing care, rehabilitation services) due to very low number of patients that incur these costs.

### Between-LHIN variation in mortality and costs

Starting with mortality, the results of the mixed models indicate that the LHIN-specific variation in mortality (represented by the variance *σ*2*u*) was statistically significant and was 10 times as low compared to non-HCU (Table 2). All covariates were statically significant with the expected signs although the impact of the number of ADG was different for HCU and non-HCU. As shown by the values of the coefficient of determination R^2^, approximately 9% the observed variation in mortality among HCU is explained by patients’ characteristics while this percentage is 18% for non-HCU.

**Table 2:** Regression results: mortality (adjusted, log scale)

Mortality		
Variables	HCU#	Non-HCU#
	Coefficient (SE)	Coefficient (SE)
Variance in mortality, *σ*2*u*	0.005 (0.003)*	0.051 (0.021)*
Intercept	−7.154 (0.087)*	−13.883 (0.201)*
Age	0.071 (0.002)*	0.109 (0.003)*
Sex, M	0.346 (0.017)*	0.392 (0.033)*
ADG	−0.04 (0.002)*	0.052 (0.004)*
Low income status	0.091 (0.02)*	0.227 (0.039)*
R^2^, %	8.8%	17.9%
LRT (Chi2 dist, *p* < 0.05)	5207.2	2800.21

# - Estimated through a mixed effects two-part model; * - estimates were statistically significant at *p* < .05; ADGs- Aggregated Diagnosis Groups; HCU- high-cost user; LHIN – Local Health Integrated Network; LRT- likelihood ratio test; R^2^- coefficients of determination; SE- Standard Error

Table 3 presents regression results for total costs among HCU and non-HCU. Since all the HCU had a contact with the healthcare system and incurred a cost, we used a GLMM to fit the data for the HCU. Results indicated that the LHIN-specific variation was small but statistically significant (*σ*2*u*2). All covariates were statistically significant too but only 1.6% (i.e. R^2^) of the observed regional variation in total costs of senior HCU was due to patient characteristics. Because 9.4% of non-HCU incurred no costs at all, we fitted a mixed effects two-part model to the non-HCU total cost data. Since two distributions generate the data, the model generates two random-effects to estimate between-LHIN variation in the probability of incurring any costs (*σ*2*u*1) and variation in costs once they were incurred (*σ*2*u*2). As shown in this table, the variation (*σ*2*u*1) among HCU in system contacts overall was 65 times as high as *σ*2*u*2_,_ both estimates statistically significant. All covariates were statistically significant in explaining each part of the model. The values of R^2^ for the non-HCU cohort indicated that 87% of the LHIN variation related to the probability of incurring a cost was explained by the covariates while only 19.7% of the variation once a cost was incurred was explained by patient characteristics of non-HCU.

**Table 3:** Regression results: total public healthcare expenditures (adjusted, log scale)

Total costs		
Variables	HCU#	Non-HCU&
	Coefficient (SE)	Coefficient (SE)
Variance in probability of incurring costs, *σ*2*u*1		0.065 (0.026)*
Variance in costs once incurred, *σ*2*u*2	0.0009 (0.0004)*	0.001 (0.001)*
Covariance between *σ*2*u*1 and *σ*2*u*2		0.006 (0.003)
Probability (costs≠0)		
Intercept		4.49 (0.16)*
Age		−0.067 (0.002)*
Sex, M		− 0.205 (0.03)*
ADG		1.018 (0.009)*
Low income status		−0.129 (0.035)*
Costs > 0		
Intercept	9.74 (0.02)*	5.946 (0.025)*
Age	0.008 (0.0002)*	0.016 (0.001)*
Sex, M	0.064 (0.003)*	0.044 (0.005)*
ADG	−0.011 (0.0004)*	0.081 (0.001)*
Low income status	0.018 (0.004)*	0.134 (0.006)*
log_theta	0.788 (0.003)*	0.473 (0.004)*
R^2^ (part 1), %		87.0%
R^2^ (part 2), %	1.6%	19.7%
LRT (Chi2 dist, *p* < 0.05)	2333.0	88,500.57

& - Estimated through GLMM; # - Estimated through a mixed effects two-part model; * - estimates were statistically significant at *p* < .05; ADGs- Aggregated Diagnosis Groups; HCU- high-cost user; LHIN – Local Health Integrated Network; Log-theta- the logarithm of the shape parameter of gamma distribution; LRT- likelihood ratio test; R^2^- coefficients of determination; SE- Standard Error

In addition to the total costs, Additional file 5A-B presents variance estimates across the cost components in both cohorts (*σ*2*u*1, where available, and *σ*2*u*2, log-scale). With the exception of the analysis of costs associated with physician visits, all cost components were analysed with two-part models. Overall, variation in incurred expenditures across cost components was higher compared with that of the total costs. Similarly, variation in the probability of positive costs was substantially greater. LHIN-specific variation in dialysis costs (both part 1 and part 2 of the model) had the highest significant values in HCU, whereas regional variation in cancer expenditures was an outlier among non-HCU. The covariates traditionally representing health care needs explained much of the observed variation in the probability of accessing healthcare: R^2^ for part 1 of the models ranged from 0.5 to 34.5% (HCU) and 6.8% to 87.0%(non-HCU). In contrast, once the costs were incurred, the role of these covariates greatly diminishes. R^2^ for part 2 ranged from 0.3 to 5.1% (HCU) and 2.7% to 19.7% (non-HCU).

### Cost-mortality relationship

**Fig. 2:**
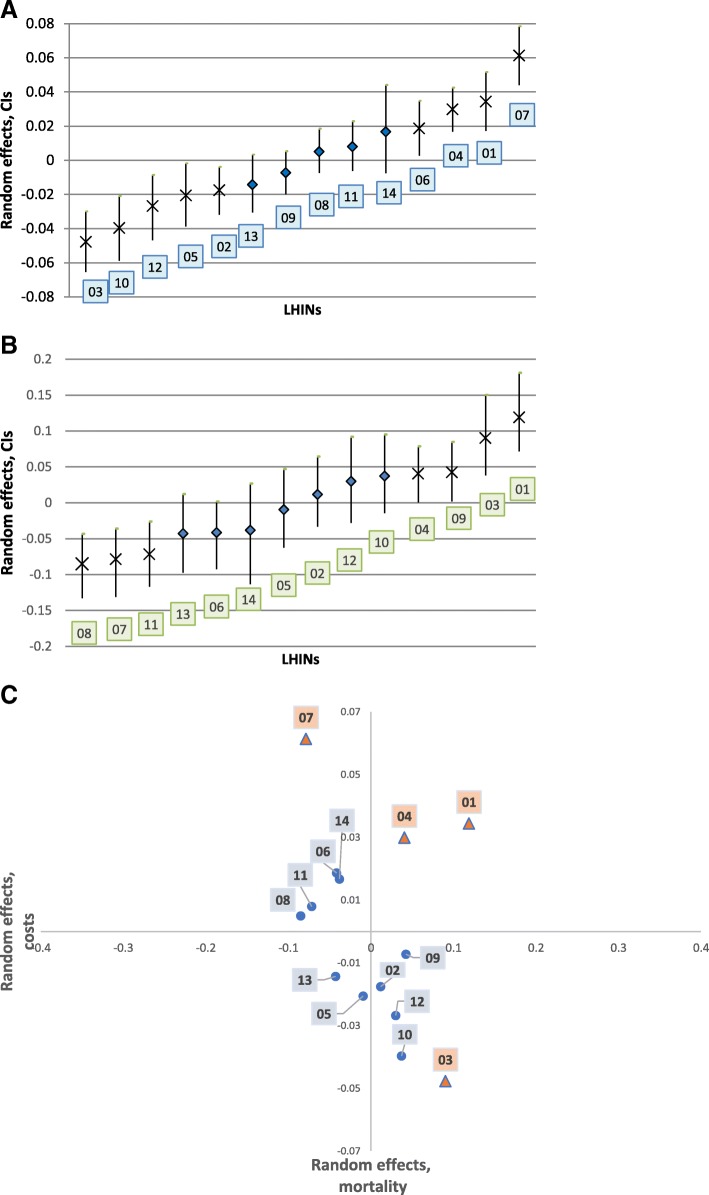
**a**: Ranking LHIN-specific random effects in total costs, HCU. Marked as X are statistically significant. **b**: Ranking LHIN-specific random effects in mortality, HCU. Marked as X are statistically significant. **c**: Cost-mortality relationship, HCU. Both total costs and mortality are adjusted for the regional factor, age, sex, ADGs, and low-income status; colored triangle indicates health district in which variation in both costs and mortality is statistically significant; 01- Erie St. Clair; 02- South West; 03- Waterloo Wellington; 04- Hamilton Niagara Haldimand Brant; 05- Central West; 06- Mississauga Halton; 07- Toronto Central; 08- Central; 09- Central East; 10- South East; 11- Champlain; 12- North Simcoe Muskoka; 13- North East; 14- North West

**Fig. 3:**
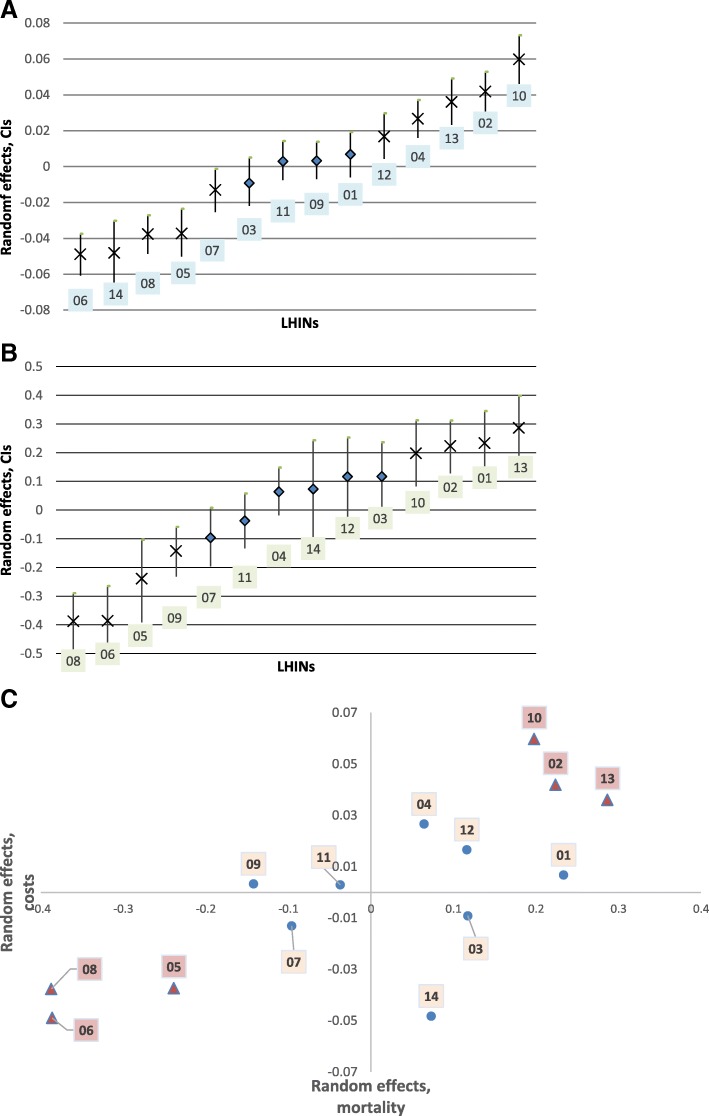
**a**: Ranking LHIN-specific random effects in total costs, non-HCU. Marked as X are statistically significant. **b**: Ranking LHIN-specific random effects in mortality, non-HCU. Marked as X are statistically significant. **c**: Cost-mortality relationship, Non-HCU. Both total costs and mortality are adjusted for the regional factor, age, sex, ADGs, and low-income status; colored triangle indicates health district in which variation in both costs and mortality is statistically significant; 01- Erie St. Clair; 02- South West; 03- Waterloo Wellington; 04- Hamilton Niagara Haldimand Brant; 05- Central West; 06- Mississauga Halton; 07- Toronto Central; 08- Central; 09- Central East; 10- South East; 11- Champlain; 12- North Simcoe Muskoka; 13- North East; 14- North West

To identify LHINs that are more efficient than others (e.g. lower spending and mortality), LHINs were ranked by random effects for total costs and mortality (Figs. 2 and 3). As shown in Figs. 2a–c presenting the random effects for each LHIN and the associated 95% CIs among HCU, there were several LHINs in which the random effects were statistically significant for mortality (Fig. 2a) and costs (Fig. 2b). When costs and mortality were combined in a cost-mortality plane (Fig. 2c), only LHINs 1, 3, 4 and 7 had the random effects significant for both total costs and mortality (marked as a triangle). Among those LHINs, none were in the lower bottom quadrant (“higher efficiency” pocket). Erie St. Claire 01 and Hamilton 04 LHINs (right upper) spend more and have a higher risk-adjusted mortality. Toronto Central 07 LHIN (left upper) has one of the lowest mortality rates, but it comes at a higher cost compared to other LHINs. In contrast, LHIN 3 had the lowest costs, but one of the highest mortality rates. For non-HCU (Fig. 3a–c), the list of LHINs with significant random effects for both total costs and mortality is broader and different from HCU. Several LHINs in close proximity to the Toronto area exhibit higher efficiency. On the opposite side, South West 02, South East 10, and North East 13 show signs of lower efficiency.

## Discussion

This is the first Canadian study to examine geographic variation in healthcare costs and mortality among senior HCU. We found approximately a 14% difference between the highest (100.2 per 1000 seniors) and lowest (88.1 per 1000 seniors) incident senior HCU rates across the LHINs in Ontario. Overall regional variation in total costs and mortality was low in both cohorts, and lower among HCU compared to non-HCU. Our results indicate that traditional drivers of costs and mortality such as age, sex, comorbidity and income play little role in explaining variation in mortality and costs among HCU. Our analyses of individual cost components revealed greater variability in accessing the healthcare system, but, once the patient enters the system, variation in costs was low. Finally, LHINs vary in their costs per mortality rate, which deserves further analysis to determine whether policies or practices followed in high performing LHINs might be usable in other LHINs.

This study’s results are important for several reasons. First, when regional variation is of interest, it is important to account for the regional factor in the model. In the literature on geographic healthcare variation, the authors seem to employ fixed effects models more often to describe variation in observed and predicted values through descriptive statistics (coefficient of variation, extremal quotient or its variations, etc.) [15, 20]. The use of mixed effects models is less frequent but provides richer information when applied. As such, in addition to controlling for the regional effect, mixed effects models directly measure regional variability by estimating a variance component. In a two-part mixed effects models such as ours, it is also possible to estimate two components of between-LHIN variation: variation in the probability of costs incurred and variation in costs once incurred. Finally, we ran a fixed effects model in parallel (results available upon a request from authors). Comparing the findings with the mixed effects models showed closely matched coefficient estimates but more narrow standard errors which is an expected difference between the fixed effects and models with random effects.

Second, exploration of regional variation across multiple cost categories among seniors has not been reported for Canadian HCU or the general population. Even internationally, this is rarely done likely due to limited availability of such data. This study results indicate that reporting variation in total spending alone hides the contribution of individual cost components. The magnitude of some cost components such as hospitalization (a mean of $13,677 among HCU) absorbs the variation of smaller components (a mean of $181 in lab costs, respectively). It is particularly so among non-HCU where healthcare expenditures are substantially lower compared to HCU. As shown here, examining regional variation as a function of total costs only would present an incomplete picture: e.g., although small regional variation in total costs, there is a much greater variation in dialysis costs among HCU. Also, comparison with non-HCU points to the fact that there is a very small number of non-HCU patients in several cost categories (e.g., mental health, rehabilitation, etc.) suggesting that incurring costs in these categories may convert a patient into an HCU.

Further, our results indicate that after adjustment, allocation of resources to seniors was similar across Ontario LHINs, more so for HCU compared to non-HCU, which is reassuring for healthcare planners. However, whether the allocation is truly equitable is unclear. Judging by the sign and CIs of coefficients in part 2 of the models, for example, the low- income status was associated with greater intensity of healthcare services across most of the cost components in both cohorts. Also, access to services may be an issue: patients with higher income status are more likely to enter the healthcare system. This is aggravated by much higher LHIN-specific variation in the probability of incurring costs. In particular, higher variation in dialysis and cancer costs may be a concern that requires further elucidation.

Finally, we have studied for the first time the relationship between costs and mortality for HCU and non-HCU across Ontario LHINs to explore health system performance from the efficiency angle. Efforts to examine the relationship between healthcare spending and outcomes, most often mortality, have been made globally applying various approaches [16, 30, 59–65]. The approach taken in our study builds on previous research that conducted hospital profiling [57, 58, 66]. As such, our results provide insight into the distribution of mortality in relation to resources spent across the Ontario LHINs by identifying districts of various cost-mortality performance. Although caution should be applied when interpreting the results of the study, e.g., variation in total costs across LHINs appears quite small, the observed differences in efficiency between health regions merit further examination to determine if health improvement could be achieved without additional healthcare spending.

### Strengths and limitations

This study has several strengths. First, the dataset contained information on all incident senior HCU in the province at the time of data collection whereas the matched non-HCU represented approximately 25% of the total senior population in the province. Second, the study examines incident HCU cases which represents a shift in the focus of HCU research dominated by studies of persistent cases (those that retain HCU status over time). Since the two populations are likely to be different, studying incident cases of HCU provides important information to inform health policies and interventions. Third, it directly estimates variation across a number of cost categories using two variance components, which has never been done in the past. Finally, to deal with the large proportion of zero costs in the data (i.e. no healthcare use), we used two part-models which have been shown to provide better estimates than models that ignore the over-representation of zeros [48] .

We note some limitations. While the study’s cost data captures public expenditures in the most expensive cost categories such as hospital admissions, physician billings, rehabilitation or home care, cost data for some components may be incomplete. For pharmaceutical care, copayment is not included in the ODB cost and, more importantly, the cost of some chemotherapy not covered by the ODB is not captured by the study, especially the costs incurred in outpatient cancer clinics [40]. The LTC costs do not include accommodation charges unless the patient’s stay is subsidized by the government. Another Canadian-based study into HCU that used the same source of administrative data but examined the entire HCU population across only 5 cost components estimated the extent of unaccounted for cost data at 7% [7]. However, since data on seniors is usually more complete, our conservative estimate is that less than 5% of government expenditures on individual health services to seniors might not have been included in this study, hence their impact on the results is close to negligible. Secondly, this study did not account for the supply side of the examined variation as the data were not available for analysis. That would require access to LHIN-based data on the number of physicians, hospital and LTC beds, HC/CCAC staff. Instead, our approach standardized for the effect of patient needs. Similarly, we did not have access to several variables which could partially explained some of the variation between LHINs (e.g. patient preferences, health behaviors, education, etc). Further, we encountered model convergence and parameter estimation issues when running models for the non-HCU’ cost components using the total non-HCU population. To address this, we re-fitted the models on a random sample of the population. Depending on the cost component, the sample size ranged from 30 to 100%.However, convergence issues in mixed effects models run on the entire population are not uncommon with very large datasets [67]. This is a limitation that in our opinion was alleviated by low discrepancy in the estimates generated by RE models compared to FE models run on the full size of the non-HCU population. Not surprisingly, as 30% of the non-HCU population is still a large enough sample (i.e., > 150,000 individuals). Finally, some may argue that a smaller unit of analysis would be preferable to evaluate regional variation. Using a smaller unit (sub-LHINs in our case) could unmask heterogeneity at the more local level. We did not have information on sub-LHINs and therefore we could not conduct these analyses. However, the choice of LHINs as the unit of analysis is supported by the fact that the boundaries of LHINs were developed with local patterns of care provision in mind [68].

## Conclusions

Risk-adjusted allocation of healthcare resources to seniors across Ontario is similar across health districts, more so for HCU than non-HCU. However, when analyzed in combination with risk-adjusted mortality, we identified important variation in the cost-mortality relationship among LHINs which needs to be further explored. The traditional drivers of costs and mortality had a weak impact on the observed variation in the outcomes among both HCU and non-HCU, but largely explained the probability of healthcare system access.

## Additional files

Additional file 1: Intra-class coefficients (ICC). Provides details on ICC calculation for costs and mortality. (DOCX 21 kb)

Additional file 2: Model specification and other statistical formulas used in statistical analysis. Provides details on model specification and formulas used in calculations. (DOCX 17 kb)

Additional file 3: A-B. Variation (by LHIN) in patient baseline individual and care characteristics, pre-incident year. The files provide information on the variation in individual and care characteristics between the 14 LHINs: for HCU (2A) and non-HCU (2B). (DOCX 30 kb)

Additional file 4: A-C. Observed and adjusted healthcare care expenditures (total and by cost component) and mortality among HCU and non-HCU, incident year. The file provides details observed and adjusted values and model fit. (DOCX 99 kb)

Additional file 5: Estimate coefficients, healthcare care expenditures among HCU and non-HCU, total costs and cost components, incident year. The file provides details on regression coefficients, including the estimates of variance components. (DOCX 49 kb)

## Abbreviations

ACGs: Johns Hopkins Adjusted Clinical Groups
ADGs: Johns Hopkins Aggregated Diagnosis Groups
BC: British Columbia, Canada
CAD: Canadian dollar
CHF: Congestive heart failure
COPD: Chronic obstructive pulmonary disease
CV: Coefficient of variation
DM: Diabetes
EDCs: John Hopkins Expanded Diagnosis Clusters
FY: Fiscal year
GLM: Generalized linear models
HCU: High-cost user
ICC: Intraclass coefficient
ICES: Institute for Clinical Evaluative Sciences
LHIN: Local Health Integration Networks
LRT: Likelihood ratio test
MI: Myocardial infarction
QGIS: Quantum geographic information system
RA: Rheumatoid arthritis
RIO: Rural Index of Ontario
SD: Standard deviation
SDD: Absolute Standardized difference
US: United States
