# Supplementary material for: Regional variation in healthcare spending and mortality among senior high-cost healthcare users in Ontario, Canada: a retrospective matched cohort study
Source: BMC Geriatr. 2018 Nov 1;18:262. doi: 10.1186/s12877-018-0952-7 (PMC6211423; doi:10.1186/s12877-018-0952-7)
Supplement: Supplementary file 1 — Intra-class coefficients (ICC). Provides details on ICC calculation for costs and mortality. (DOCX 21 kb) [file 12877_2018_952_MOESM1_ESM.docx]

**Appendix 1: Intra-class coefficients (ICC)**

1. Costs

| **HCU** | **Parameters** | **Total** | **Hosp** | **MD** | **HC** | **ODB** | **ED** | **Lab** | **LTC** | **MH** | **Rehab** | **Dialysis** | **Cancer** | **CCC** |
| --- | --- | --- | --- | --- | --- | --- | --- | --- | --- | --- | --- | --- | --- | --- |
|  | **Random intercept, subject=LHIN** | 0.0012 | 0.0013 | 0.0170 | 0.0073 | 0.0015 | 0.0018 | 0.0110 | 0.0010 | 0.01966 | 0.0084 | 0.3000 | 0.0236 | 0.0358 |
|  | **Residual** | 0.4596 | 0.7708 | 0.3929 | 0.9227 | 1.1425 | 0.4521 | 0.6176 | 0.7640 | 0.7687 | 0.013 | 0.126 | 0.021 | 0.037 |
|  | **ICC, %** | 0.26% | 0.17% | 4.15% | 0.79% | 0.13% | 0.40% | 1.74% | 0.13% | 2.49% | 1.26% | 12.61% | 2.05% | 3.70% |
|  | p-value (random intercept) | 0.001 | 0.027 | 0.007 | 0.008 | 0.026 | 0.02 | 0.006 | 0.36 | 0.98 | 0.656 | 2.080 | 1.124 | 0.93 |
|  |  |  |  |  |  |  |  |  |  |  |  |  |  |  |
| **Non-HCU** | **Random intercept, subject=LHIN** | 0.0010 | 0.0029 | 0.0140 | 0.0140 | 0.0017 | 0.0018 | 0.0087 | not defined | not defined | 0.0003 | 0.4641 | 0.2264 | not defined |
|  | p-value (random intercept) | 0.012 | 0.024 | 0.005 | 0.008 | 0.001 | 0.02 | 0.005 |  |  | 0.47 | 0.09 | 0.019 |  |
|  | **Residual** | 0.7110 | 0.5554 | 0.5448 | 0.5860 | 1.2780 | 0.4521 | 0.5288 |  |  | 0.0029 | 0.2694 | 0.8596 |  |
|  | **ICC, %** | 0.14% | 0.53% | 2.51% | 2.33% | 0.13% | 0.40% | 1.61% |  |  | 8.29% | 63.27% | 20.85% |  |

ICC=estimate of random intercept/residual+ estimate of random intercept (%)

Abbreviations: Hosp- hospitalization costs, MD-physician costs, HC-home care, ODB-Outpatient Drug Benefit, ED- emergency department, LTC-long-term care, MH-mental health, CCC-complex continuing care

1. Mortality

| **Parameters** | **HCU** | **Non-HCU** |
| --- | --- | --- |
| **Random intercept, subject=LHIN** | 0.004159 | 0.04208 |
| p-value (random intercept) | 0.017 | 0.009 |
| **Residual** | 3.29 | 3.29 |
| **ICC, %** | 0.126% | 1.263% |
