# Supplementary material for: Regional variation in healthcare spending and mortality among senior high-cost healthcare users in Ontario, Canada: a retrospective matched cohort study
Source: BMC Geriatr. 2018 Nov 1;18:262. doi: 10.1186/s12877-018-0952-7 (PMC6211423; doi:10.1186/s12877-018-0952-7)
Supplement: Supplementary file 2 — Model specification and other statistical formulas used in statistical analysis. Provides details on model specification and formulas used in calculations. (DOCX 17 kb) [file 12877_2018_952_MOESM2_ESM.docx]

**Appendix 2: Model specification and other statistical formulas used in statistical analysis**

1. General equation

y_ij_=(ꞵ0+u0_j_) + ∑ꞵ_ij_X_ij_+e_ij_

where $yij$ –is the outcome (costs or mortality) in patient i from LHINj; $ꞵ0$ – the provincial mean; $u0j$ – is the random effect for each LHIN that is assumed $u0j\sim N\left( 0,\sigma2u \right)$; $ꞵij$ - are the fixed effects of individual level characteristics; $Xij$ - is the vector of covariates at the individual level; $eij$ - is the residual error.

1. Coefficient of determination (R2)
   1. Binary outcome (e.g., mortality)

$$R2=\frac{\sigma2fe}{\sigma2u+ \sigma2fe+ \pi2/3}$$

- 1. Continuous outcome with gamma distribution (i.e., costs)

$$R2=\frac{\sigma2fe}{\sigma2u+ \sigma2fe+\ln(1+\frac{1}{v})}$$

where $\sigma2fe$- variance explained by fixed effects; $\sigma2u$- variance explained by random effects (LHINs); $v$- the shape parameter from gamma GLM
