# Supplementary material for: Regional variation in healthcare spending and mortality among senior high-cost healthcare users in Ontario, Canada: a retrospective matched cohort study
Source: BMC Geriatr. 2018 Nov 1;18:262. doi: 10.1186/s12877-018-0952-7 (PMC6211423; doi:10.1186/s12877-018-0952-7)
Supplement: Supplementary file 3 — A-B. Variation (by LHIN) in patient baseline individual and care characteristics, pre-incident year. The files provide information on the variation in individual and care characteristics between the 14 LHINs: for HCU (2A) and non-HCU (2B). (DOCX 30 kb) [file 12877_2018_952_MOESM3_ESM.docx]

Additional files 3A-B. **Variation (by LHIN) in patient baseline individual and care characteristics, pre-incident year**

**A: HCU**

|  | **LHIN** | **Age (%)** | | | **Sex (F, %)** | **Rurality (% of urban)** | **Low income senior (%)** | **Number of ADGs, mean per patient** | **Proportion with chronic conditions (%)** | | | **Number of physicians involved in care, mean per patient** | **Seen by a geriatrician (%)** | **Number of prescription drugs, mean per patient** | **Acute inpatient admissions (%)** |
| --- | --- | --- | --- | --- | --- | --- | --- | --- | --- | --- | --- | --- | --- | --- | --- |
|  |  | 66-74 | 75-84 | ≥85 |  |  |  |  | Malignant neoplasms | Common chronic conditions* | Mental health# |  |  |  |  |
| 1 | Erie St. Clair | 39.9 | 39.4 | 20.8 | 53.6 | 32.7 | 13.6 | 10.6 | 33.1 | 65.3 | 40.1 | 8.3 | 0.5 | 9.0 | 3.5 |
| 2 | South West | 38.8 | 39.7 | 21.6 | 53.5 | 39.5 | 14.7 | 9.9 | 37.3 | 59.2 | 35.6 | 7.5 | 2.6 | 8.0 | 4.1 |
| 3 | Waterloo Wellington | 37.9 | 40.4 | 21.7 | 53.4 | 75.9 | 14.3 | 9.6 | 33.0 | 58.3 | 34.2 | 7.3 | 4.1 | 7.9 | 3.4 |
| 4 | Hamilton Niagara | 38.1 | 40.1 | 21.7 | 53.8 | 76.8 | 14.1 | 10.2 | 32.7 | 60.1 | 36.9 | 8.1 | 3.5 | 8.4 | 3.5 |
| 5 | Central West | 44.5 | 38.9 | 16.6 | 51.3 | 91.1 | 28.1 | 10.4 | 26.8 | 63.2 | 40.6 | 8.3 | 3.3 | 9.2 | 3.5 |
| 6 | Mississauga Halton | 38.4 | 39.8 | 21.8 | 52.6 | 100.0 | 19.5 | 10.5 | 31.1 | 59.0 | 40.4 | 8.5 | 5.0 | 8.6 | 3.6 |
| 7 | Toronto Central | 36.5 | 39.2 | 24.3 | 55.4 | 100.0 | 25.7 | 10.4 | 32.8 | 57.0 | 45.1 | 8.5 | 3.6 | 8.3 | 2.8 |
| 8 | Central | 36.3 | 40.6 | 23.1 | 53.1 | 87.4 | 23.9 | 10.8 | 31.8 | 59.6 | 42.4 | 8.9 | 3.9 | 8.8 | 2.9 |
| 9 | Central East | 38.0 | 40.8 | 21.3 | 52.4 | 60.1 | 21.2 | 10.3 | 31.8 | 62.6 | 37.3 | 8.6 | 2.6 | 8.8 | 2.8 |
| 10 | South East | 42.4 | 39.6 | 18.0 | 50.7 | 33.8 | 14.8 | 9.6 | 31.5 | 60.1 | 33.6 | 8.1 | 1.2 | 8.1 | 2.7 |
| 11 | Champlain | 39.3 | 38.3 | 22.4 | 53.5 | 64.4 | 14.9 | 10.1 | 33.4 | 58.4 | 39.6 | 8.4 | 1.3 | 8.1 | 3.3 |
| 12 | North Simcoe Muskoka | 43.0 | 39.8 | 17.3 | 52.1 | 22.6 | 13.7 | 9.9 | 34.9 | 60.6 | 35.5 | 7.4 | 2.2 | 7.9 | 4.5 |
| 13 | North East | 42.6 | 41.3 | 16.1 | 51.2 | 25.1 | 16.3 | 9.8 | 28.5 | 63.9 | 35.9 | 7.2 | 1.5 | 8.5 | 6.0 |
| 14 | North West | 39.8 | 40.3 | 19.9 | 51.1 | 56.3 | 11.1 | 9.9 | 29.9 | 61.6 | 28.9 | 7.9 | 4.3 | 7.9 | 6.4 |
|  |  |  |  |  |  |  |  |  |  |  |  |  |  |  |  |
| **CV** | | **6.3%** | **2%** | **12.3%** | **2.5%** | **44.7%** | **29.7%** | **3.7%** | **8.1%** | **4.0%** | **11.0%** | **6.5%** | **47.8%** | **5.2%** | **30.0%** |

ADGs- Aggregated Diagnosis Groups; CV- Coefficient of Variation; HCU- high-cost user; *ICES- derived common chronic conditions (CHF- congestive heart failure; COPD- chronic obstructive pulmonary disease; DM- diabetes, MI- myocardial infarction, RA- rheumatoid arthritis); LHIN – Local Health Integrated Network; SD- Standard Deviation; # includes any of mental health conditions among Expanded Diagnosis Clusters (PSY01-12)

**B: Non-HCU**

|  | **LHIN** | **Age (%)** | | | **Sex (F, %)** | **Rurality (% of urban)** | **Low income senior (%)** | **Comorbidity (# of ADGs), mean per patient** | **Proportion with chronic conditions (%)** | | | **Number of MDs involved in care, mean per patient** | **Seen by a geriatrician (%)** | **Number of prescription drugs, mean per patient** | **Acute inpatient admissions (%)** |
| --- | --- | --- | --- | --- | --- | --- | --- | --- | --- | --- | --- | --- | --- | --- | --- |
|  |  | 66-74 | 75-84 | ≥85 |  |  |  |  | Malignant neoplasms | Common chronic conditions* | Mental health# |  |  |  |  |
| 1 | Erie St. Clair | 39.9 | 39.4 | 20.8 | 53.6 | 34.6 | 13.1 | 8.4 | 25.6 | 49.2 | 31.2 | 6.0 | 0.3 | 6.2 | 2.1 |
| 2 | South West | 38.8 | 39.7 | 21.6 | 53.5 | 40.2 | 12.9 | 8.1 | 29.3 | 44.9 | 26.4 | 5.4 | 1.1 | 5.6 | 2.3 |
| 3 | Waterloo Wellington | 37.9 | 40.4 | 21.7 | 53.4 | 76.8 | 13.4 | 7.6 | 24.7 | 42.3 | 24.4 | 5.2 | 1.9 | 5.3 | 1.9 |
| 4 | Hamilton Niagara | 38.1 | 40.1 | 21.7 | 53.8 | 75.9 | 13.2 | 8.3 | 25.8 | 44.1 | 27.9 | 6.0 | 1.4 | 5.8 | 2.0 |
| 5 | Central West | 44.5 | 38.9 | 16.6 | 51.3 | 93.3 | 27.5 | 7.7 | 17.3 | 46.1 | 27.0 | 5.5 | 1.1 | 5.9 | 1.7 |
| 6 | Mississauga Halton | 38.4 | 39.8 | 21.8 | 52.6 | 100.0 | 18.4 | 7.7 | 20.4 | 42.5 | 27.1 | 5.5 | 1.9 | 5.3 | 1.7 |
| 7 | Toronto Central | 36.5 | 39.2 | 24.3 | 55.4 | 100.0 | 25.2 | 7.5 | 21.5 | 42.1 | 29.9 | 5.4 | 1.3 | 5.2 | 1.4 |
| 8 | Central | 36.3 | 40.6 | 23.1 | 53.1 | 90.0 | 24.6 | 7.9 | 20.5 | 43.4 | 28.3 | 5.8 | 1.4 | 5.6 | 1.5 |
| 9 | Central East | 38.0 | 40.8 | 21.3 | 52.4 | 67.1 | 22.2 | 7.9 | 22.6 | 46.8 | 26.6 | 5.8 | 1.1 | 5.9 | 1.4 |
| 10 | South East | 42.4 | 39.6 | 18.0 | 50.7 | 33.2 | 12.6 | 7.9 | 24.1 | 44.2 | 25.0 | 6.0 | 0.6 | 5.5 | 1.8 |
| 11 | Champlain | 39.3 | 38.3 | 22.4 | 53.5 | 69.1 | 14.3 | 7.9 | 24.1 | 42.4 | 28.3 | 5.8 | 0.5 | 5.3 | 1.6 |
| 12 | North Simcoe Muskoka | 43.0 | 39.8 | 17.3 | 52.1 | 21.7 | 11.9 | 8.1 | 28.2 | 45.5 | 27.3 | 5.5 | 1.0 | 5.5 | 2.5 |
| 13 | North East | 42.6 | 41.3 | 16.1 | 51.2 | 26.2 | 15.6 | 7.9 | 21.9 | 48.4 | 25.9 | 5.3 | 0.5 | 5.9 | 3.4 |
| 14 | North West | 39.8 | 40.3 | 19.9 | 51.1 | 50.4 | 10.8 | 7.6 | 21.5 | 46.0 | 20.7 | 5.5 | 1.9 | 5.1 | 3.4 |
|  |  |  |  |  |  |  |  |  |  |  |  |  |  |  |  |
| **CV** | | **6.3%** | **2.0%** | **12.3%** | **2.5%** | **44.7%** | **33.6%** | **3.5%** | **13.8%** | **5.1%** | **9.3%** | **4.6%** | **48.4%** | **5.7%** | **31.8%** |

ADGs- Aggregated Diagnosis Groups; CV- Coefficient of Variation; HCU- high-cost user; *ICES- derived common chronic conditions (CHF- congestive heart failure; COPD- chronic obstructive pulmonary disease; DM- diabetes, MI- myocardial infarction, RA- rheumatoid arthritis); LHIN – Local Health Integrated Network; SD- Standard Deviation; # includes any of mental health conditions among Expanded Diagnosis Clusters (PSY01-12)
