# Supplementary material for: Regional variation in healthcare spending and mortality among senior high-cost healthcare users in Ontario, Canada: a retrospective matched cohort study
Source: BMC Geriatr. 2018 Nov 1;18:262. doi: 10.1186/s12877-018-0952-7 (PMC6211423; doi:10.1186/s12877-018-0952-7)
Supplement: Supplementary file 4 — A-C. Observed and adjusted healthcare care expenditures (total and by cost component) and mortality among HCU and non-HCU, incident year. The file provides details observed and adjusted values and model fit. (DOCX 99 kb) [file 12877_2018_952_MOESM4_ESM.docx]

Additional files 4A-B **Observed and adjusted healthcare care expenditures (total and by cost component) and mortality among HCUs and non-HCUs, incident year**

**A: HCU**

Models are adjusted for random effects (RE), age, sex, ADGs, and low-income status; AIC/BIC- Akaike/Bayesian information criterion; CV- Coefficient of variation; HCU- high-cost user, LL- loglikelihood; ODB-Outpatient Drug Benefit plan; SD- standard deviation;

| **LHIN** | | **Total costs** | | | **Hospital admission** | | | **Physician** | | | **Home care** | | |
| --- | --- | --- | --- | --- | --- | --- | --- | --- | --- | --- | --- | --- | --- |
|  |  | Obs | RE | Adj | Obs | RE | Adj | Obs | RE | Adj | Obs | RE | Adj |
|  | Proportion of zero values (%) | 0.0 |  |  | 11.7 |  |  | 0.03 |  |  | 41.6 |  |  |
| 1 | Erie St. Clair | 30,559 | 30,518 | 30,510 | 14,204 | 14,165 | 14,175 | 4,152 | 4,151 | 4,138 | 1,771 | 1,772 | 1,780 |
| 2 | South West | 29,136 | 29,151 | 29,204 | 13,620 | 13,634 | 13,654 | 3,575 | 3,576 | 3,566 | 1,991 | 1,989 | 1,988 |
| 3 | Waterloo Wellington | 28,305 | 28,373 | 28,459 | 12,798 | 12,864 | 12,925 | 3,852 | 3,853 | 3,844 | 1,800 | 1,800 | 1,798 |
| 4 | Hamilton Niagara | 30,576 | 30,557 | 30,546 | 13,539 | 13,534 | 13,553 | 4,171 | 4,171 | 4,169 | 2,156 | 2,153 | 2,129 |
| 5 | Central West | 28,773 | 28,820 | 28,813 | 13,441 | 13,469 | 13,439 | 4,875 | 4,872 | 4,883 | 1,570 | 1,574 | 1,565 |
| 6 | Mississauga Halton | 30,251 | 30,227 | 30,175 | 13,291 | 13,319 | 13,305 | 4,655 | 4,654 | 4,675 | 1,460 | 1,464 | 1,479 |
| 7 | Toronto Central | 31,813 | 31,737 | 31,649 | 13,536 | 13,511 | 13,411 | 4,703 | 4,702 | 4,720 | 1,916 | 1,912 | 1,917 |
| 8 | Central | 29,785 | 29,781 | 29,771 | 13,164 | 13,174 | 13,146 | 4,851 | 4,850 | 4,867 | 1,618 | 1,619 | 1,618 |
| 9 | Central East | 29,487 | 29,490 | 29,481 | 13,286 | 13,298 | 13,290 | 4,371 | 4,371 | 4,371 | 1,551 | 1,553 | 1,556 |
| 10 | South East | 28,434 | 28,495 | 28,523 | 13,003 | 13,022 | 13,043 | 3,861 | 3,861 | 3,852 | 2,001 | 1,996 | 1,981 |
| 11 | Champlain | 29,929 | 29,921 | 29,926 | 13,652 | 13,632 | 13,638 | 4,047 | 4,047 | 4,048 | 1,579 | 1,581 | 1,587 |
| 12 | North Simcoe Muskoka | 28,647 | 28,708 | 28,752 | 13,774 | 13,796 | 13,820 | 4,124 | 4,124 | 4,118 | 1,742 | 1,742 | 1,731 |
| 13 | North East | 29,109 | 29,133 | 29,165 | 14,833 | 14,795 | 14,834 | 3,344 | 3,346 | 3,343 | 1,663 | 1,665 | 1,664 |
| 14 | North West | 30,238 | 30,165 | 30,187 | 15,338 | 15,130 | 15,171 | 3,171 | 3,176 | 3,188 | 1,810 | 1,808 | 1,815 |
|  | **Mean** | 29,646 | 29,648 | 29,654 | 13,677 | 13,667 | 13,672 | 4,125 | 4,125 | 4,127 | 1,759 | 1,759 | 1,758 |
|  | **SD** | 992.8 | 948.9 | 913.6 | 694.6 | 639.7 | 651.7 | 536.4 | 534.8 | 541.0 | 201.8 | 199.3 | 193.2 |
|  | **CV** | 3.3 | 3.2 | 3.1 | 5.1 | 4.7 | 4.8 | 13.0 | 13.0 | 13.1 | 11.5 | 11.3 | 11.0 |
|  | -2 Log Likelihood | 3925362 | 3925073 | 3922740 | 3426248 | 3425853 | 3422126 | 3221763 | 3216335 | 3209694 | 2090097 | 2087329 | 2067947 |
|  | AIC (smaller is better) | 3925366 | 3925079 | 3922754 | 3426254 | 3425865 | 3422154 | 3221767 | 3216341 | 3209708 | 2090103 | 2087341 | 2067975 |
|  | BIC (smaller is better) | 3925386 | 3925080 | 3922758 | 3426284 | 3425869 | 3422162 | 3221787 | 3216343 | 3209712 | 2090133 | 2087344 | 2067984 |
|  | LRT (Chi2 dist, p<0.05) |  | 289.2 | 2333.0 |  | 394.6 | 3727.4 |  | 5428.2 | 6640.9 |  | 2768.4 | 19381.3 |

| **LHIN** | | **ODB** | | | **Emergency department** | | | **Mental health** | | | **Lab** | | |
| --- | --- | --- | --- | --- | --- | --- | --- | --- | --- | --- | --- | --- | --- |
|  |  | Obs | RE | Adj | Obs | RE | Adj | Obs | RE | Adj | Obs | RE | Adj |
|  | Proportion of zero values (%) | 0.9 |  |  | 24.3 |  |  | 99.0 |  |  | 14.4 |  |  |
| 1 | Erie St. Clair | 2,553 | 2,549 | 2,559 | 895 | 894 | 893 | 194 | 207 | 207 | 177 | 177 | 177 |
| 2 | South West | 2,301 | 2,306 | 2,302 | 865 | 866 | 864 | 324 | 313 | 313 | 149 | 150 | 150 |
| 3 | Waterloo Wellington | 2,401 | 2,406 | 2,395 | 823 | 824 | 823 | 345 | 313 | 312 | 199 | 199 | 199 |
| 4 | Hamilton Niagara | 2,535 | 2,533 | 2,544 | 840 | 840 | 842 | 207 | 212 | 210 | 198 | 198 | 197 |
| 5 | Central West | 2,527 | 2,524 | 2,532 | 823 | 823 | 824 | 151 | 178 | 180 | 209 | 208 | 208 |
| 6 | Mississauga Halton | 2,395 | 2,396 | 2,394 | 825 | 826 | 825 | 266 | 261 | 257 | 214 | 214 | 214 |
| 7 | Toronto Central | 2,283 | 2,283 | 2,270 | 852 | 852 | 855 | 351 | 333 | 332 | 170 | 170 | 171 |
| 8 | Central | 2,422 | 2,423 | 2,415 | 816 | 816 | 818 | 232 | 235 | 237 | 221 | 220 | 220 |
| 9 | Central East | 2,505 | 2,503 | 2,505 | 835 | 835 | 835 | 186 | 195 | 193 | 212 | 212 | 212 |
| 10 | South East | 2,590 | 2,582 | 2,587 | 863 | 863 | 862 | 269 | 262 | 257 | 164 | 164 | 164 |
| 11 | Champlain | 2,566 | 2,563 | 2,571 | 888 | 887 | 886 | 356 | 341 | 341 | 162 | 162 | 162 |
| 12 | North Simcoe Muskoka | 2,466 | 2,464 | 2,467 | 859 | 860 | 859 | 286 | 275 | 280 | 184 | 184 | 184 |
| 13 | North East | 2,474 | 2,472 | 2,478 | 915 | 915 | 912 | 214 | 223 | 229 | 145 | 145 | 146 |
| 14 | North West | 2,103 | 2,144 | 2,131 | 1,099 | 1,087 | 1,089 | 161 | 204 | 197 | 128 | 129 | 129 |
|  | **Mean** | 2,437 | 2,439 | 2,439 | 871 | 871 | 870 | 253 | 254 | 253 | 181 | 181 | 181 |
|  | **SD** | 134.5 | 124.7 | 132.0 | 71.9 | 69.0 | 69.1 | 71.5 | 54.3 | 54.5 | 29.0 | 28.8 | 28.5 |
|  | **CV** | 5.5 | 5.1 | 5.4 | 8.3 | 7.9 | 7.9 | 28.2 | 21.4 | 21.5 | 16.0 | 15.9 | 15.7 |
|  | -2 Log Likelihood | 3088995 | 3088721 | 3084420 | 2295081 | 2294521 | 2287076 | 58709 | 58656 | 58378 | 2049266 | 2043455 | 2037745 |
|  | AIC (smaller is better) | 3089001 | 3088733 | 3084448 | 2295087 | 2294533 | 2287104 | 58715 | 58668 | 58406 | 2049272 | 2043467 | 2037773 |
|  | BIC (smaller is better) | 3089031 | 3088737 | 3084457 | 2295118 | 2294537 | 2287113 | 58745 | 58672 | 58415 | 2049302 | 2043471 | 2037781 |
|  | LRT (Chi2 dist, p<0.05) |  | 273.7 | 4300.8 |  | 560.6 | 7445.3 |  | 53.0 | 278.2 |  | 5811.1 | 5710.6 |

| **LHIN** | | **Dialysis** | | | **Cancer** | | | **LTC** | | | | **CCC** | | | **Rehabilitation** | | |
| --- | --- | --- | --- | --- | --- | --- | --- | --- | --- | --- | --- | --- | --- | --- | --- | --- | --- |
|  |  | Obs | RE | Adj | Obs | RE | Adj | Obs | RE | Adj | Obs | | RE | Adj | Obs | RE | Adj |
|  | Proportion of zero values (%) | 99.3 |  |  | 90.1 |  |  | 93.4 |  |  | 95.4 | |  |  | 92.5 |  |  |
| 1 | Erie St. Clair | 158 | 132 | 128 | 1,189 | 1,190 | 1,199 | 1,062 | 1,064 | 1,064 | 1,093 | | 1,085 | 1,079 | 1,849 | 1,832 | 1,819 |
| 2 | South West | 83 | 92 | 91 | 1,355 | 1,291 | 1,319 | 1,254 | 1,245 | 1,248 | 758 | | 769 | 771 | 994 | 1,003 | 1,003 |
| 3 | Waterloo Wellington | 99 | 101 | 102 | 1,536 | 1,516 | 1,527 | 1,165 | 1,135 | 1,141 | 1,073 | | 1,079 | 1,086 | 950 | 959 | 958 |
| 4 | Hamilton Niagara | 133 | 126 | 125 | 1,291 | 1,276 | 1,285 | 1,066 | 1,061 | 1,063 | 1,760 | | 1,756 | 1,757 | 1,048 | 1,040 | 1,035 |
| 5 | Central West | 146 | 128 | 132 | 1,207 | 1,222 | 1,231 | 844 | 884 | 871 | 527 | | 550 | 547 | 1,026 | 1,052 | 1,053 |
| 6 | Mississauga Halton | 81 | 84 | 85 | 1,474 | 1,468 | 1,462 | 793 | 815 | 819 | 1,188 | | 1,178 | 1,175 | 2,015 | 1,987 | 1,971 |
| 7 | Toronto Central | 58 | 78 | 78 | 1,125 | 1,173 | 1,131 | 735 | 744 | 756 | 2,211 | | 2,178 | 2,171 | 1,785 | 1,792 | 1,804 |
| 8 | Central | 121 | 115 | 112 | 1,181 | 1,192 | 1,178 | 921 | 921 | 924 | 950 | | 950 | 952 | 1,581 | 1,585 | 1,593 |
| 9 | Central East | 99 | 101 | 102 | 1,206 | 1,233 | 1,227 | 1,101 | 1,070 | 1,065 | 1,010 | | 1,012 | 1,013 | 1,821 | 1,817 | 1,828 |
| 10 | South East | 125 | 113 | 115 | 1,118 | 1,184 | 1,206 | 1,048 | 982 | 963 | 791 | | 811 | 810 | 719 | 737 | 732 |
| 11 | Champlain | 56 | 76 | 75 | 1,298 | 1,271 | 1,268 | 987 | 984 | 991 | 725 | | 718 | 718 | 1,709 | 1,702 | 1,698 |
| 12 | North Simcoe Muskoka | 92 | 98 | 99 | 1,254 | 1,239 | 1,281 | 1,021 | 1,071 | 1,066 | 593 | | 604 | 604 | 777 | 793 | 794 |
| 13 | North East | 85 | 94 | 98 | 1,140 | 1,159 | 1,183 | 1,063 | 1,095 | 1,087 | 773 | | 769 | 772 | 821 | 809 | 802 |
| 14 | North West | 197 | 153 | 150 | 1,256 | 1,235 | 1,276 | 724 | 815 | 812 | 1,687 | | 1,664 | 1,659 | 858 | 866 | 869 |
|  | **Mean** | 109 | 106 | 107 | 1,259 | 1,261 | 1,269 | 985 | 992 | 991 | 1,081 | | 1,080 | 1,080 | 1,282 | 1,284 | 1,283 |
|  | **SD** | 39.6 | 22.2 | 21.7 | 125.2 | 106.3 | 108.6 | 160.2 | 141.0 | 140.4 | 488.0 | | 475.5 | 473.8 | 476.5 | 467.4 | 467.5 |
|  | **CV** | 36.2 | 20.9 | 20.3 | 9.9 | 8.4 | 8.6 | 16.3 | 14.2 | 14.2 | 45.1 | | 44.0 | 43.9 | 37.2 | 36.4 | 36.4 |
|  | -2 Log Likelihood | 38786 | 37751 | 37613 | 477492 | 476322 | 472853 | 332125 | 331899 | 322971 | 244232 | | 243120 | 240498 | 379798 | 378292 | 376573 |
|  | AIC (smaller is better) | 38792 | 37763 | 37641 |  | 476334 | 472881 |  | 331911 | 322999 | 244238 | | 243132 | 240526 | 379804 | 378304 | 376601 |
|  | BIC (smaller is better) | 38822 | 37767 | 37650 |  | 476337 | 472890 |  | 331915 | 323008 | 244268 | | 243136 | 240535 | 379835 | 378308 | 376610 |
|  | LRT (Chi2 dist, p<0.05) |  | 1035.2 | 138.1 |  | 1170.7 | 3468.5 |  | 225.9 | 8928.5 |  | | 1111.6 | 2622.1 |  | 1506.3 | 1719.0 |

**B: Non-HCU**

| **LHIN** | | **Total costs (30% sample)** | | | **Hospital admission (50% sample)** | | | **Physician (100% sample)** | | | **Home care (75% sample)** | | |
| --- | --- | --- | --- | --- | --- | --- | --- | --- | --- | --- | --- | --- | --- |
|  |  | Obs | RE | Adj | Obs | RE | Adj | Obs | RE | Adj | Obs | RE | Adj |
|  | Proportion of zero values (%) | 9.4 |  |  | 85.9 |  |  | 10.50 |  |  | 92.5 |  |  |
| 1 | Erie St. Clair | 2,558 | 2,553 | 2,601 | 248 | 248 | 249 | 811 | 811 | 818 | 135 | 135 | 135 |
| 2 | South West | 2,593 | 2,590 | 2,621 | 263 | 262 | 260 | 739 | 739 | 749 | 148 | 148 | 147 |
| 3 | Waterloo Wellington | 2,408 | 2,418 | 2,431 | 242 | 242 | 242 | 772 | 772 | 785 | 146 | 145 | 146 |
| 4 | Hamilton Niagara | 2,633 | 2,629 | 2,658 | 241 | 240 | 239 | 843 | 843 | 848 | 139 | 139 | 138 |
| 5 | Central West | 2,348 | 2,350 | 2,348 | 188 | 188 | 191 | 830 | 830 | 821 | 93 | 94 | 93 |
| 6 | Mississauga Halton | 2,266 | 2,273 | 2,260 | 192 | 193 | 193 | 807 | 807 | 803 | 82 | 83 | 84 |
| 7 | Toronto Central | 2,323 | 2,319 | 2,321 | 142 | 143 | 144 | 798 | 799 | 789 | 130 | 130 | 131 |
| 8 | Central | 2,414 | 2,411 | 2,414 | 173 | 173 | 176 | 849 | 849 | 839 | 120 | 120 | 120 |
| 9 | Central East | 2,444 | 2,442 | 2,458 | 206 | 207 | 208 | 829 | 829 | 827 | 134 | 134 | 134 |
| 10 | South East | 2,565 | 2,564 | 2,589 | 266 | 267 | 267 | 769 | 769 | 781 | 135 | 134 | 133 |
| 11 | Champlain | 2,451 | 2,449 | 2,458 | 211 | 211 | 212 | 735 | 735 | 740 | 118 | 118 | 119 |
| 12 | North Simcoe Muskoka | 2,561 | 2,561 | 2,592 | 280 | 279 | 276 | 820 | 819 | 825 | 88 | 89 | 89 |
| 13 | North East | 2,514 | 2,515 | 2,534 | 332 | 332 | 330 | 631 | 631 | 644 | 121 | 121 | 120 |
| 14 | North West | 2,217 | 2,262 | 2,273 | 312 | 303 | 302 | 517 | 520 | 528 | 128 | 128 | 127 |
|  | **Mean** | 2,450 | 2,453 | 2,468 | 235 | 235 | 235 | 768 | 768 | 771 | 123 | 123 | 123 |
|  | **SD** | 128.0 | 120.3 | 134.0 | 53.5 | 52.3 | 50.9 | 92.3 | 91.4 | 87.6 | 21.1 | 20.5 | 20.3 |
|  | **CV** | 5.2 | 4.9 | 5.4 | 22.7 | 22.3 | 21.7 | 12.0 | 11.9 | 11.4 | 17.2 | 16.7 | 16.6 |
|  | -2 Log Likelihood | 2644680 | 2642366 | 2553865 | 834313 | 832874 | 821522 | 1905951 | 1902381 | 1832307 | 708186 | 706833 | 674824 |
|  | AIC (smaller is better) | 2644686 | 2642378 | 2553893 | 834319 | 832886 | 821550 | 1905957 | 1902393 | 1832335 | 708192 | 706845 | 674852 |
|  | BIC (smaller is better) | 2644716 | 2642381 | 2553902 | 834350 | 832890 | 821559 | 1905987 | 1902397 | 1832344 | 708224 | 706849 | 674861 |
|  | LRT (Chi2 dist, p<0.05) |  | 2314.1 | 88500.6 |  | 1438.6 | 11352.1 |  | 3570.0 | 70073.9 |  | 1352.4 | 32008.9 |

| **LHIN** | | **ODB (50% sample)** | | | **Emergency department (40% sample)** | | | **Mental health** | | | **Lab (100% sample)** | | |
| --- | --- | --- | --- | --- | --- | --- | --- | --- | --- | --- | --- | --- | --- |
|  |  | Obs | RE | Adj | Obs | RE | Adj | Obs | RE | Adj | Obs | RE | Adj |
|  | Proportion of zero values (%) | 15.6 |  |  | 79.6 |  |  |  |  |  | 31.1 |  |  |
| 1 | Erie St. Clair | 931 | 927 | 935 | 103 | 103 | 103 |  |  |  | 98 | 98 | 99 |
| 2 | South West | 864 | 865 | 873 | 118 | 119 | 119 |  |  |  | 88 | 88 | 89 |
| 3 | Waterloo Wellington | 830 | 832 | 834 | 94 | 93 | 93 |  |  |  | 105 | 105 | 105 |
| 4 | Hamilton Niagara | 901 | 900 | 905 | 101 | 101 | 102 |  |  |  | 114 | 114 | 114 |
| 5 | Central West | 844 | 841 | 846 | 68 | 69 | 69 |  |  |  | 107 | 107 | 106 |
| 6 | Mississauga Halton | 810 | 810 | 807 | 74 | 74 | 74 |  |  |  | 115 | 115 | 114 |
| 7 | Toronto Central | 761 | 763 | 759 | 74 | 74 | 74 |  |  |  | 98 | 98 | 98 |
| 8 | Central | 823 | 823 | 819 | 74 | 74 | 74 |  |  |  | 117 | 117 | 116 |
| 9 | Central East | 897 | 895 | 898 | 85 | 85 | 85 |  |  |  | 114 | 114 | 114 |
| 10 | South East | 859 | 861 | 866 | 111 | 112 | 111 |  |  |  | 93 | 93 | 94 |
| 11 | Champlain | 830 | 830 | 827 | 99 | 99 | 99 |  |  |  | 93 | 93 | 93 |
| 12 | North Simcoe Muskoka | 869 | 870 | 871 | 107 | 108 | 107 |  |  |  | 101 | 101 | 102 |
| 13 | North East | 916 | 914 | 925 | 133 | 133 | 133 |  |  |  | 83 | 83 | 86 |
| 14 | North West | 772 | 785 | 792 | 154 | 150 | 150 |  |  |  | 70 | 71 | 71 |
|  | **Mean** | 851 | 851 | 854 | 100 | 100 | 99 |  |  |  | 100 | 100 | 100 |
|  | **SD** | 50.8 | 48.2 | 51.6 | 24.6 | 23.9 | 23.8 |  |  |  | 13.6 | 13.4 | 12.7 |
|  | **CV** | 6.0 | 5.7 | 6.0 | 24.6 | 24.0 | 23.9 |  |  |  | 13.6 | 13.4 | 12.7 |
|  | -2 Log Likelihood | 3748630 | 3744757 | 3628439 | 814041 | 811982 | 798286 |  |  |  | 1237071 | 1234478 | 1202377 |
|  | AIC (smaller is better) | 3748636 | 3744769 | 3628467 | 814047 | 811994 | 798314 |  |  |  | 1237077 | 1234490 | 1202405 |
|  | BIC (smaller is better) | 3748667 | 3744773 | 3628476 | 814078 | 811998 | 798322 | 58745 | 58672 | 58415 | 1237106 | 1234494 | 1202414 |
|  | LRT (Chi2 dist, p<0.05) |  | 3872.9 | 116317.5 |  | 2059.4 | 13696.6 |  | 53.0 | 278.2 |  | 2592.5 | 32100.9 |

| **LHIN** | | **Dialysis (50% sample)** | | | **Cancer (100% sample)** | | | **LTC** | | | | **CCC** | | | **Rehabilitation** | | |
| --- | --- | --- | --- | --- | --- | --- | --- | --- | --- | --- | --- | --- | --- | --- | --- | --- | --- |
|  |  | Obs | RE | Adj | Obs | RE | Adj | Obs | RE | Adj | Obs | | RE | Adj | Obs | RE | Adj |
|  | Proportion of zero values (%) | 99.9 |  |  | 99.5 |  |  |  |  |  |  | |  |  |  |  |  |
| 1 | Erie St. Clair | - | 0.01 | 0.01 | 1 | 1 | 1 |  |  |  |  | |  |  |  |  |  |
| 2 | South West | - | 0.01 | 0.01 | 2 | 2 | 2 |  |  |  |  | |  |  |  |  |  |
| 3 | Waterloo Wellington | 0.03 | 0.05 | 0.05 | 15 | 14 | 14 |  |  |  |  | |  |  |  |  |  |
| 4 | Hamilton Niagara | 0.22 | 0.16 | 0.17 | 4 | 4 | 4 |  |  |  |  | |  |  |  |  |  |
| 5 | Central West | 0.23 | 0.24 | 0.24 | 3 | 3 | 3 |  |  |  |  | |  |  |  |  |  |
| 6 | Mississauga Halton | 2.54 | 2.53 | 2.52 | 8 | 8 | 8 |  |  |  |  | |  |  |  |  |  |
| 7 | Toronto Central | - | 0.01 | 0.01 | 2 | 2 | 2 |  |  |  |  | |  |  |  |  |  |
| 8 | Central | 0.08 | 0.08 | 0.09 | 2 | 2 | 2 |  |  |  |  | |  |  |  |  |  |
| 9 | Central East | 0.03 | 0.03 | 0.03 | 4 | 4 | 4 |  |  |  |  | |  |  |  |  |  |
| 10 | South East | - | 0.01 | 0.01 | 3 | 2 | 2 |  |  |  |  | |  |  |  |  |  |
| 11 | Champlain | - | 0.01 | 0.01 | 2 | 2 | 2 |  |  |  |  | |  |  |  |  |  |
| 12 | North Simcoe Muskoka | 0.02 | 0.03 | 0.03 | 2 | 2 | 2 |  |  |  |  | |  |  |  |  |  |
| 13 | North East | - | 0.01 | 0.01 | 1 | 1 | 1 |  |  |  |  | |  |  |  |  |  |
| 14 | North West | 0.95 | 0.86 | 0.84 | 4 | 3 | 3 |  |  |  |  | |  |  |  |  |  |
|  | **Mean** | 0.29 | 0.29 | 0.29 | 4 | 4 | 4 |  |  |  |  | |  |  |  |  |  |
|  | **SD** | 0.7 | 0.7 | 0.7 | 3.5 | 3.5 | 3.5 |  |  |  |  | |  |  |  |  |  |
|  | **CV** | 236.0 | 237.5 | 237.0 | 91.8 | 92.7 | 92.8 |  |  |  |  | |  |  |  |  |  |
|  | -2 Log Likelihood | 7202 | 6163 | 5952 | 67710 | 63939 | 62850 |  |  |  |  | |  |  |  |  |  |
|  | AIC (smaller is better) | 7208 | 6175 | 5980 | 67716 | 63951 | 62878 |  |  |  |  | |  |  |  |  |  |
|  | BIC (smaller is better) | 7240 | 6178 | 5989 | 67750 | 63955 | 62887 |  |  |  |  | |  |  |  |  |  |
|  | LRT (Chi2 dist, p<0.05) |  | 1039.9 | 210.3 |  | 3771.5 | 1088.6 |  |  |  |  | |  |  |  |  |  |

**C. Mortality**

| **LHIN** | | **HCU** | | | **Non-HCU** | | |
| --- | --- | --- | --- | --- | --- | --- | --- |
|  |  | Obs | RE | Adj | Obs | RE | Adj |
|  | Proportion of zero values (%) |  |  |  |  |  |  |
| 1 | Erie St. Clair | 115.7 | 113.4 | 113.1 | 9.8 | 9.6 | 9.6 |
| 2 | South West | 106.8 | 106.4 | 106.6 | 9.8 | 9.7 | 9.7 |
| 3 | Waterloo Wellington | 118.2 | 115.1 | 115.9 | 8.7 | 8.6 | 8.6 |
| 4 | Hamilton Niagara | 109.0 | 108.5 | 108.6 | 8.4 | 8.4 | 8.4 |
| 5 | Central West | 97.5 | 99.1 | 97.8 | 5.2 | 5.5 | 5.4 |
| 6 | Mississauga Halton | 100.7 | 101.3 | 101.4 | 5.0 | 5.3 | 5.3 |
| 7 | Toronto Central | 100.9 | 101.4 | 102.2 | 7.4 | 7.5 | 7.5 |
| 8 | Central | 98.3 | 99.0 | 99.2 | 5.5 | 5.6 | 5.6 |
| 9 | Central East | 110.6 | 109.9 | 110.2 | 6.8 | 6.9 | 6.9 |
| 10 | South East | 106.1 | 105.7 | 105.2 | 8.7 | 8.6 | 8.6 |
| 11 | Champlain | 98.6 | 99.3 | 99.5 | 7.6 | 7.6 | 7.6 |
| 12 | North Simcoe Muskoka | 103.3 | 103.5 | 102.3 | 8.0 | 7.9 | 7.8 |
| 13 | North East | 95.2 | 97.1 | 96.1 | 9.2 | 9.1 | 9.0 |
| 14 | North West | 98.1 | 100.7 | 100.5 | 7.9 | 7.8 | 7.8 |
|  | **Mean** | 104.2 | 104.3 | 104.2 | 7.7 | 7.7 | 7.7 |
|  | **SD** | 7.1 | 5.7 | 6.0 | 1.6 | 1.5 | 1.5 |
|  | **CV** | 6.8 | 5.4 | 5.7 | 20.6 | 18.8 | 19.1 |
|  | -2 Log Likelihood | 117793.8 | 117753.8 | 112546.6 | 47190.7 | 47070.6 | 44270.4 |
|  | AIC (smaller is better) | 117795.8 | 117757.8 | 112558.6 | 47192.7 | 47074.6 | 44282.4 |
|  | BIC (smaller is better) | 117805.9 | 117759.1 | 112562.4 | 47203.9 | 47075.9 | 44286.2 |
|  | LRT (Chi2 dist, p<0.05) |  | 40 | 5207.2 |  | 120.12 | 2800.21 |
