# Supplementary material for: Regional variation in healthcare spending and mortality among senior high-cost healthcare users in Ontario, Canada: a retrospective matched cohort study
Source: BMC Geriatr. 2018 Nov 1;18:262. doi: 10.1186/s12877-018-0952-7 (PMC6211423; doi:10.1186/s12877-018-0952-7)
Supplement: Supplementary file 5 — Estimate coefficients, healthcare care expenditures among HCU and non-HCU, total costs and cost components, incident year. The file provides details on regression coefficients, including the estimates of variance components. (DOCX 49 kb) [file 12877_2018_952_MOESM5_ESM.docx]

Appendix 5 **Estimate coefficients, healthcare care expenditures in HCUs, total costs and cost components, incident year**

**A: HCU**

$\text{σ2u}\text{1}$ – variance of the probability of incurring costs; $\text{σ2u2}$ – variance of the costs incurred; R2- coefficient of determination (for part 1 and part 2 of two-part models);

|  | **Total costs** | | | **Hospital admission** | | | **Physician** | | | **Home care** | | | | **ODB** | | | |
| --- | --- | --- | --- | --- | --- | --- | --- | --- | --- | --- | --- | --- | --- | --- | --- | --- | --- |
|  | Coeff | SE | P-value | Coeff | SE | P-value | Coeff | SE | P-value | Coeff | SE | P-value | Coeff | | SE | P-value |  |
| $\text{σ2u1}$ |  |  |  | 0.022 | 0.009 | 0.0297 |  |  |  | 0.052 | 0.020 | 0.0221 | 0.044 | | 0.020 | 0.0447 |  |
| $\text{σ2u2}$ | 0.001 | 0.000 | 0.0264 | 0.001 | 0.001 | 0.0348 | 0.018 | 0.007 | 0.0205 | 0.007 | 0.003 | 0.0232 | 0.003 | | 0.001 | 0.0283 |  |
| **Covariance (u1 and u2)** |  |  |  | 0.003 | 0.002 | 0.122 |  |  |  | -0.002 | 0.005 | 0.6936 | 0.007 | | 0.004 | 0.0949 |  |
| **p(costs)=0; Intercept** |  |  |  | 4.212 | 0.089 | <.0001 |  |  |  | -5.310 | 0.083 | <.0001 | 4.732 | | 0.277 | <.0001 |  |
| Age |  |  |  | -0.028 | 0.001 | <.0001 |  |  |  | 0.077 | 0.001 | <.0001 | -0.023 | | 0.003 | <.0001 |  |
| Sex, M |  |  |  | 0.182 | 0.015 | <.0001 |  |  |  | -0.387 | 0.010 | <.0001 | -0.436 | | 0.054 | <.0001 |  |
| ADG |  |  |  | -0.002 | 0.002 | 0.2725 |  |  |  | -0.008 | 0.001 | <.0001 | 0.252 | | 0.007 | <.0001 |  |
| Low – income status |  |  |  | -0.062 | 0.019 | 0.0071 |  |  |  | 0.125 | 0.014 | <.0001 | -0.168 | | 0.064 | 0.023 |  |
| **p(costs)>0; Intercept** | 9.739 | 0.019 | <.0001 | 9.170 | 0.026 | <.0001 | 9.245 | 0.039 | <.0001 | 6.234 | 0.038 | <.0001 | 7.925 | | 0.031 | <.0001 |  |
| Age | 0.008 | 0.000 | <.0001 | 0.008 | 0.000 | <.0001 | -0.013 | 0.000 | <.0001 | 0.023 | 0.000 | <.0001 | -0.006 | | 0.000 | <.0001 |  |
| Sex, M | 0.064 | 0.003 | <.0001 | 0.125 | 0.005 | <.0001 | 0.104 | 0.003 | <.0001 | -0.053 | 0.006 | <.0001 | 0.047 | | 0.005 | <.0001 |  |
| ADG | -0.011 | 0.000 | <.0001 | -0.020 | 0.001 | <.0001 | 0.005 | 0.000 | <.0001 | -0.002 | 0.001 | 0.0306 | 0.027 | | 0.001 | <.0001 |  |
| Low – income status | 0.018 | 0.004 | 0.0009 | 0.022 | 0.006 | 0.0031 | -0.050 | 0.004 | <.0001 | 0.037 | 0.007 | 0.0004 | 0.221 | | 0.007 | <.0001 |  |
| log_theta | 0.788 | 0.003 | <.0001 | 0.267 | 0.003 | <.0001 | 0.969 | 0.003 | <.0001 | 0.126 | 0.004 | <.0001 | -0.113 | | 0.003 | <.0001 |  |
| **R2 (part 1)** |  |  |  | 0.5% |  |  | 34.5% |  |  | 10.7% |  |  | 24.5% | |  |  |  |
| **R2 (part 2)** | 1.6% |  |  | 2.2% |  |  | 4.0% |  |  | 5.1% |  |  | 2.3% | |  |  |  |

|  | **Emergency department** | | | **Mental health** | | | **Lab** | | | **Dialysis** | | | **Cancer** | | |
| --- | --- | --- | --- | --- | --- | --- | --- | --- | --- | --- | --- | --- | --- | --- | --- |
|  | Coeff | SE | P-value | Coeff | SE | P-value | Coeff | SE | P-value | Coeff | SE | P-value | Coeff | SE | P-value |
| $\text{σ2u1}$ | 0.032 | 0.013 | 0.0271 | 0.033 | 0.016 | 0.06 | 0.160 | 0.061 | 0.0219 | 0.392 | 0.153 | 0.0248 | 0.063 | 0.024 | 0.0229 |
| $\text{σ2u2}$ | 0.002 | 0.001 | 0.0284 | 0.018 | 0.010 | 0.1064 | 0.011 | 0.004 | 0.0225 | 0.357 | 0.141 | 0.0257 | 0.020 | 0.008 | 0.0257 |
| **Covariance (u1 and u2)** | 0.007 | 0.003 | 0.0405 | -0.005 | 0.009 | 0.6213 | 0.023 | 0.013 | 0.0932 | -0.342 | 0.139 | 0.0298 | -0.034 | 0.013 | 0.0263 |
| **p(costs)=0; Intercept** | -3.015 | 0.078 | <.0001 | 0.653 | 0.271 | 0.0328 | 1.525 | 0.130 | <.0001 | -4.776 | 0.358 | <.0001 | 2.015 | 0.112 | <.0001 |
| Age | 0.051 | 0.001 | <.0001 | 0.052 | 0.003 | <.0001 | -0.007 | 0.001 | <.0001 | -0.013 | 0.004 | 0.0062 | -0.053 | 0.001 | <.0001 |
| Sex, M | 0.082 | 0.011 | <.0001 | 0.271 | 0.049 | 0.0001 | -0.035 | 0.014 | 0.0271 | 0.557 | 0.060 | <.0001 | 0.075 | 0.016 | 0.0006 |
| ADG | 0.022 | 0.001 | <.0001 | -0.006 | 0.006 | 0.3366 | 0.082 | 0.002 | <.0001 | 0.028 | 0.007 | 0.0026 | -0.020 | 0.002 | <.0001 |
| Low – income status | 0.263 | 0.016 | <.0001 | -0.231 | 0.062 | 0.003 | -0.150 | 0.018 | <.0001 | 0.354 | 0.073 | 0.0004 | -0.300 | 0.024 | <.0001 |
| **p(costs)>0; Intercept** | 6.404 | 0.023 | <.0001 | 9.764 | 0.244 | <.0001 | 4.942 | 0.035 | <.0001 | 10.667 | 0.508 | <.0001 | 11.366 | 0.101 | <.0001 |
| Age | 0.006 | 0.000 | <.0001 | 0.007 | 0.003 | 0.0484 | 0.001 | 0.000 | 0.0024 | -0.005 | 0.006 | 0.3919 | -0.026 | 0.001 | <.0001 |
| Sex, M | 0.031 | 0.004 | <.0001 | 0.042 | 0.043 | 0.3564 | 0.046 | 0.004 | <.0001 | -0.083 | 0.087 | 0.3585 | 0.145 | 0.016 | <.0001 |
| ADG | 0.016 | 0.000 | <.0001 | -0.017 | 0.005 | 0.0038 | 0.029 | 0.001 | <.0001 | -0.039 | 0.011 | 0.0035 | -0.007 | 0.002 | 0.0068 |
| Low – income status | 0.046 | 0.005 | <.0001 | 0.047 | 0.054 | 0.4024 | 0.006 | 0.005 | 0.3123 | -0.028 | 0.104 | 0.7948 | -0.115 | 0.024 | 0.0005 |
| log_theta | 0.810 | 0.004 | <.0001 | 0.272 | 0.031 | <.0001 | 0.499 | 0.003 | <.0001 | -0.721 | 0.034 | <.0001 | -0.069 | 0.009 | <.0001 |
| **R2 (part 1)** | 5.0% |  |  | 4.6% |  |  | 3.3% |  |  | 3.0% |  |  | 5.7% |  |  |
| **R2 (part 2)** | 1.7% |  |  | 1.6% |  |  | 3.0% |  |  | 2.9% |  |  | 4.9% |  |  |

|  | **LTC** | | | **CCC** | | | **Rehabilitation** | | |
| --- | --- | --- | --- | --- | --- | --- | --- | --- | --- |
|  | Coeff | SE | P-value | Coeff | SE | P-value | Coeff | SE | P-value |
| $\text{σ2u1}$ | 0.044 | 0.017 | 0.0262 | 0.126 | 0.049 | 0.0239 | 0.140 | 0.054 | 0.02302 |
| $\text{σ2u2}$ | 0.001 | 0.001 | 0.2826 | 0.026 | 0.011 | 0.0311 | 0.006 | 0.003 | 0.000268 |
| **Covariance (u1 and u2)** | -0.004 | 0.003 | 0.2017 | 0.001 | 0.016 | 0.9579 | -0.002 | 0.008 | -0.01988 |
| **p(costs)=0; Intercept** | -11.062 | 0.129 | <.0001 | -8.430 | 0.159 | <.0001 | -5.480 | 0.139 | -5.783 |
| Age | 0.111 | 0.001 | <.0001 | 0.072 | 0.002 | <.0001 | 0.041 | 0.001 | 0.03817 |
| Sex, M | -0.412 | 0.021 | <.0001 | -0.095 | 0.024 | 0.0019 | -0.279 | 0.019 | -0.3202 |
| ADG | -0.039 | 0.003 | <.0001 | -0.037 | 0.003 | <.0001 | -0.022 | 0.002 | -0.0268 |
| Low – income status | 0.163 | 0.023 | <.0001 | 0.059 | 0.028 | 0.06 | -0.101 | 0.023 | -0.1516 |
| **p(costs)>0; Intercept** | 9.295 | 0.098 | <.0001 | 9.877 | 0.131 | <.0001 | 9.322 | 0.079 | 9.1486 |
| Age | 0.005 | 0.001 | 0.001 | 0.003 | 0.001 | 0.0551 | 0.006 | 0.001 | 0.003755 |
| Sex, M | -0.044 | 0.017 | 0.0234 | -0.001 | 0.023 | 0.9624 | 0.142 | 0.015 | 0.1101 |
| ADG | -0.007 | 0.002 | 0.0058 | -0.008 | 0.003 | 0.0126 | -0.005 | 0.002 | -0.00834 |
| Low – income status | -0.016 | 0.018 | 0.3996 | 0.032 | 0.027 | 0.2505 | 0.037 | 0.018 | -0.00206 |
| log_theta | 0.312 | 0.012 | <.0001 | 0.028 | 0.014 | 0.0695 | 0.433 | 0.011 | 0.4081 |
| **R2 (part 1)** | 19.5% |  |  | 8.8% |  |  | 3.7% |  |  |
| **R2 (part 2)** | 0.5% |  |  | 0.3% |  |  | 1.3% |  |  |

**B Non-HCU**

Note: mixed effects models in the following cost categories did not converge among senior non-HCUs: mental health, complex continuing care, long-term care, and rehabilitation services

|  | **Total costs** | | | **Hospital admission** | | | **Physician** | | | **Home care** | | | | **ODB** | | | |
| --- | --- | --- | --- | --- | --- | --- | --- | --- | --- | --- | --- | --- | --- | --- | --- | --- | --- |
|  | Coeff | SE | P-value | Coeff | SE | P-value | Coeff | SE | P-value | Coeff | SE | P-value | Coeff | | SE | P-value |  |
| $\text{σ2u1}$ | 0.065 | 0.026 | 0.03 | 0.048 | 0.019 | 0.03 | 0.117 | 0.046 | 0.03 | 0.035 | 0.014 | 0.03 | 0.088 | | 0.034 | 0.03 |  |
| $\text{σ2u2}$ | 0.001 | 0.001 | 0.04 | 0.003 | 0.002 | 0.05 | 0.014 | 0.005 | 0.03 | 0.015 | 0.006 | 0.03 | 0.002 | | 0.001 | 0.03 |  |
| **Covariance (u1 and u2)** | 0.006 | 0.003 | 0.06 | 0.005 | 0.004 | 0.17 | 0.02 | 0.012 | 0.13 | -0.009 | 0.007 | 0.21 | 0.007 | | 0.004 | 0.08 |  |
| **p(costs)=0; Intercept** | 4.49 | 0.16 | <.0001 | -1.534 | 0.086 | <.0001 | 3.639 | 0.167 | <.0001 | -13.64 | 0.096 | <.0001 | 0.694 | | 0.111 | <.0001 |  |
| Age | -0.067 | 0.002 | <.0001 | -0.019 | 0.001 | <.0001 | -0.057 | 0.002 | <.0001 | 0.125 | 0.001 | <.0001 | -0.023 | | 0.001 | <.0001 |  |
| Sex, M | -0.205 | 0.03 | <.0001 | 0.254 | 0.012 | <.0001 | -0.163 | 0.029 | <.0001 | -0.438 | 0.014 | <.0001 | -0.192 | | 0.016 | <.0001 |  |
| ADG | 1.018 | 0.009 | <.0001 | 0.125 | 0.002 | <.0001 | 0.805 | 0.007 | <.0001 | 0.138 | 0.002 | <.0001 | 0.556 | | 0.003 | <.0001 |  |
| Low – income status | -0.129 | 0.035 | 0.003 | -0.179 | 0.017 | <.0001 | -0.096 | 0.034 | 0.02 | 0.213 | 0.016 | <.0001 | 0.041 | | 0.019 | 0.05 |  |
| **p(costs)>0; Intercept** | 5.946 | 0.025 | <.0001 | 6.325 | 0.046 | <.0001 | 5.719 | 0.039 | <.0001 | 6.44 | 0.065 | <.0001 | 5.257 | | 0.029 | <.0001 |  |
| Age | 0.016 | 0.001 | <.0001 | 0.017 | 0.001 | <.0001 | 0.005 | 0.001 | <.0001 | 0.013 | 0.001 | <.0001 | 0.014 | | 0.001 | <.0001 |  |
| Sex, M | 0.044 | 0.005 | <.0001 | -0.021 | 0.008 | 0.02 | 0.019 | 0.005 | 0 | -0.092 | 0.01 | <.0001 | 0.118 | | 0.005 | <.0001 |  |
| ADG | 0.081 | 0.001 | <.0001 | -0.022 | 0.002 | <.0001 | 0.074 | 0.001 | <.0001 | -0.005 | 0.002 | 0 | 0.055 | | 0.001 | <.0001 |  |
| Low – income status | 0.134 | 0.006 | <.0001 | 0.044 | 0.012 | 0 | -0.059 | 0.006 | <.0001 | 0.055 | 0.011 | 0 | 0.424 | | 0.007 | <.0001 |  |
| log_theta | 0.473 | 0.004 | <.0001 | 0.61 | 0.007 | <.0001 | 0.736 | 0.004 | <.0001 | 0.543 | 0.008 | <.0001 | -0.19 | | 0.003 | <.0001 |  |
| **R2 (part 1)** | 87.0% |  |  | 9.8% |  |  | 79.9% |  |  | 28.9% |  |  | 64.9% | |  |  |  |
| **R2 (part 2)** | 19.7% |  |  | 2.7% |  |  | 17.5% |  |  | 2.7% |  |  | 9.2% | |  |  |  |

|  | **Emergency department** | | | **Lab** | | | **Dialysis** | | | **Cancer** | | |
| --- | --- | --- | --- | --- | --- | --- | --- | --- | --- | --- | --- | --- |
|  | Coeff | SE | P-value | Coeff | SE | P-value | Coeff | SE | P-value | Coeff | SE | P-value |
| $\text{σ2u1}$ | 0.092 | 0.035 | 0.03 | 0.073 | 0.028 | 0.03 | 7.305 | 4.439 | 0.13 | 1.325 | 0.508 | 0.03 |
| $\text{σ2u2}$ | 0.002 | 0.001 | 0.06 | 0.008 | 0.004 | 0.03 | 0.547 | 0.39 | 0.19 | 0.305 | 0.122 | 0.03 |
| **Covariance (u1 and u2)** | 0.006 | 0.004 | 0.14 | 0.007 | 0.007 | 0.37 | -1.196 | 1.22 | 0.35 | -0.583 | 0.235 | 0.03 |
| **p(costs)=0; Intercept** | -3.668 | 0.102 | <.0001 | 1.185 | 0.102 | <.0001 | -12.49 | 1.169 | <.0001 | -7.341 | 0.385 | <.0001 |
| Age | 0.015 | 0.001 | <.0001 | -0.028 | 0.001 | <.0001 | 0.007 | 0.01 | 0.46 | -0.003 | 0.003 | 0.34 |
| Sex, M | -0.08 | 0.012 | <.0001 | -0.052 | 0.014 | 0 | 0.881 | 0.136 | <.0001 | 0.27 | 0.042 | <.0001 |
| ADG | 0.142 | 0.002 | <.0001 | 0.248 | 0.002 | <.0001 | 0.169 | 0.015 | <.0001 | 0.154 | 0.005 | <.0001 |
| Low – income status | 0.047 | 0.016 | 0.01 | -0.008 | 0.018 | 0.69 | 0.898 | 0.141 | <.0001 | -0.186 | 0.061 | 0.01 |
| **p(costs)>0; Intercept** | 5.259 | 0.036 | <.0001 | 4.187 | 0.036 | <.0001 | 5.519 | 0.628 | <.0001 | 6.827 | 0.262 | <.0001 |
| Age | 0.009 | 0.001 | <.0001 | 0.006 | 0.001 | <.0001 | 0.009 | 0.007 | 0.17 | 0.004 | 0.003 | 0.18 |
| Sex, M | -0.012 | 0.007 | 0.09 | 0.028 | 0.005 | <.0001 | -0.04 | 0.083 | 0.64 | 0.032 | 0.037 | 0.4 |
| ADG | 0.02 | 0.001 | <.0001 | 0.039 | 0.001 | <.0001 | 0.004 | 0.013 | 0.78 | -0.005 | 0.006 | 0.35 |
| Low – income status | 0.063 | 0.009 | <.0001 | 0.007 | 0.007 | 0.34 | -0.009 | 0.085 | 0.92 | -0.001 | 0.053 | 1 |
| log_theta | 0.84 | 0.007 | <.0001 | 0.706 | 0.005 | <.0001 | 1.039 | 0.087 | <.0001 | 0.265 | 0.026 | <.0001 |
| **R2 (part 1)** | 10.5% |  |  | 27.7% |  |  | 6.8% |  |  | 8.6% |  |  |
| **R2 (part 2)** | 3.1% |  |  | 5.5% |  |  | 3.1% |  |  | 0.6% |  |  |
